# Tropical fruits in the Mediterranean Basin: current research status, priorities, and knowledge gaps. A systematic review

**DOI:** 10.3389/fpls.2026.1817537

**Published:** 2026-06-05

**Authors:** Mark Massaad, Dario Scuderi, Francesco Andolina, Serena Bellitti, Angelo Buscaglia, Giovanni Gugliuzza, Guido La Sala, Matteo Mezzano, Giulia Salsi, Ilenia Tinebra, Vittorio Farina

**Affiliations:** 1 Department of Agricultural, Food and Forest Sciences (SAAF), University of Palermo, Palermo, Italy; 2 Independent researcher, Palermo, Italy; 3 Consiglio per la ricerca in agricoltura e l'analisi dell'economia agraria (CREA) – Research Centre for Plant Protection and Certification, c/o Department of Agricultural, Food and Forestry Sciences, University of Palermo, Palermo, Italy

**Keywords:** avocado, cherimoya, dragon fruit, litchi, mango, papaya, passion fruit

## Abstract

Mediterranean Basin has recently emerged as a viable region for tropical fruit cultivation, as reflected by the expansion and increase of dedicated agricultural areas. However, research on these species remains scattered, limited, and not always easily accessible. In this context, evidence-based synthesis is fundamental to assess the current research status and to identify knowledge gaps. In this study, we conduct a comprehensive systematic review to highlight research trends on major tropical fruit crops (mango, avocado, papaya, guava, litchi, dragon fruit, passion fruit, and cherimoya) across countries of the Mediterranean basin. The objective is to summarise key findings and pinpoint gaps that require further investigation. A final dataset of 517 publications was obtained following a PRISMA search protocol. Information, including studied species, varieties, year, geographic location, and thematic area, was retrieved and quantitatively analysed. Subsequently, a summary was compiled for each species based on the most relevant findings. Avocado (n = 200) and mango (n = 166) were the most studied species, whereas litchi, passionfruit, and guava were the least studied. Over the past 90 years, an average of five articles were published annually, with an increase to 17 per year in the last decade. A geographical research bias was evident, with most publications originating from the European part of the Mediterranean. Spain, Italy, and Egypt accounted for the highest number of publications, while Cyprus and Algeria had the fewest. Most studies were focused on pests and pathology, whereas ecosystem services and ethnobotany were the least explored thematic areas. Although grey literature and national reports may not have been fully captured due to indexing limitations, this review serves as both a compendium and a baseline for future research on tropical fruit cultivation in the Mediterranean Basin.

## 1 Introduction

Tropical fruits are cultivated and considered native to regions between the Tropic of Cancer and the Tropic of Capricorn, where warm and humid climatic conditions prevail. Most of these fruits are edible and appreciated for their distinctive taste (Baiyeri and Ugese, 2011). Tropical and subtropical plants, particularly, fruits trees, have been introduced to the Mediterranean countries on many different occasions and through multiple ports of entry. Over time, these species were brought either through botanical gardens or private collections. One of the earliest tropical trees introduced to the region was the avocado, documented in a botanical garden in Valencia, Spain, as early as 1601 (Popenoe and Zentmyer, 1963). According to official sources, mango trees were introduced from India to Egypt in 1825 (Yadav and Singh, 2017). It was is only at the end of the 20^th^ century that social changes, with a globalization of the consumers’ tastes and dietary habits, have significantly increased the demand for fresh exotic fruits in the Mediterranean basin (Claval and Jourdain-Annequin, 2018; CBI, 2020). Climate change and increased trade have converged to offer farmers in the Mediterranean countries the opportunity to grow species native to different climatic zones (Scuderi et al., 2023; Noordam, 2024). To meet the increasing and widespread demand, single growers and small cooperatives invested in the cultivation of these new crops, despite its high economic and environmental risks (Sayadi and Calatrava, 2009).

Nowadays, a total of ca. 300.000 ha of land across the Mediterranean is dedicated to the cultivation of tropical crops, according to FAOSTAT database under the categories avocadoes; mangoes; guavas, papaya and mangosteens (FAO, 2022). Over the last 20 years, agricultural surface dedicated for tropical fruit production increased on average by 81% among all Mediterranean countries. Egypt is the country with the largest cultivation area with 165.000 ha, followed by Spain (51.000 ha) and Israel (18500 ha). This increase in cultivated areas was more marked in countries where tropical species were recently introduced. Italy, for example, now ranks 4th regarding cultivated area, while there were no reports of cultivation of these crops in 2005. This trend is reflected in the economic value of this lucrative segment within the agricultural sector. In Lebanon, avocado exports grew from $533,000 in 2012 to $3.3 million by 2020, signalling a rising focus on high-value crops (MOET, 2022).

However, tropical crops cultivation remains somehow unfamiliar for growers of the Mediterranean basin and is still facing many constraints and challenges. In fact, the introduction of new crops can be the cause of diffusion of new pests (Sailer, 1983). Moreover, cultivation of non-native species to a new environment requires specific agricultural management and may have significant impact on the environment and natural resources (Byrne and Stone, 2011). Research institutions and universities in this region are expected to play an important role in guiding growers, investigate the best agricultural practices and assessing the adaptability of the species in relation to the peculiar climatic conditions.

In this work, we aim to address several key questions related to tropical fruit crop research in Mediterranean countries;

1. Explore and describe the temporal evolution of research on tropical fruit crops in the Mediterranean Basin over time;
2. Quantify the main focal points of the literature (i.e. crop species, research themes, and geographical distribution), and identify potential biases in research coverage;
3. Identify the most studied species and topics, as well as those that remain underrepresented;
4. Summarize the main findings reported across studies and highlight current challenges and research gaps to support future research and development.

This work will serve as a comprehensive overview of the current state of knowledge on tropical fruit cultivation in the Mediterranean basin and as a baseline for future works and studies.

## 2 Materials and methods

### 2.1 Literature research

To generate the largest possible database of publications conducted on tropical fruit cultivation across the Mediterranean basin, a systematic search was performed using the Scopus and Web of Science (WOS) databases, in March 2024 following PRISMA protocol (Moher et al., 2009). Publications including research articles, reports and books were considered, published in English, French, Turkish, Spanish, Italian and Arabic from 1932 till 2024. The following combination of keywords: “species_name” AND “country_name”, were used as search strings and applied to the title, keywords and abstract fields. As for species we considered the following species: avocado, cherimoya/annona, dragon fruit, guava, litchi, mango, papaya, and passionfruit, using both their common and scientific and Latin names. These species were selected based on their presence in Mediterranean basin. For each species, a separate search was conducted combined with the following Mediterranean countries: Albania, Algeria, Bosnia and Herzegovina, Croatia, Cyprus, Egypt, France, Greece, Israel, Italy, Lebanon, Libya, Malta, Monaco, Montenegro, Morocco, Palestine, Slovenia, Spain, Syria, Tunisia, and Turkey. Additionally, we included the largest Mediterranean islands: Sicily, Sardinia, Corsica, Crete, Euboea, Mallorca, Lesbos, and Rhodes, as well as “Mediterranean Basin” to ensure comprehensive coverage.

### 2.2 Screening and eligibility criteria

Once research strings were done, publications from both sources were merged and combined into a single database. All duplicated publications were removed. A first screening was done based on the title and abstract. Then a full text screening was conducted for all eligible publications, according to Moher et al. (2009). Studies were included when they met the two following inclusion criteria: 1) article must be conducted in one of the selected countries; 2) article must be covering one of the selected species; and no restriction was applied to the publication research topic except those related to health and medicine. Studies conducted outside the Mediterranean region or conducted on no selected species were excluded.

All eligible publications were then subjected to a thorough full text screening. For each publication the following data - when available - were retrieved: year of publication, type of paper (review, article, conference papers, report, book etc.), language, continent (Africa, Asia and Europe), country, city, studied species, studied varieties (Appendix A), studied part of plants (fruit, branch, flower, root, and the whole plant), research topic, used methods, pre - or post-harvest, study area (lab, field or both), cropping conditions (open air, greenhouse, shading nets). Publications were categorized into ten main thematic areas (**Table 1**) based on their primary research focus and broader scope of impact. To ensure coherence and reduce subjectivity, the categorization was conducted collaboratively by all authors. Pre- and post-harvest was assigned based on the timing of the study and depending on if the analysis were done before or after harvesting. We also examined whether the study was done in the field, laboratory or both.

**Table 1:** List of research topics identified in the database and standardized main thematic areas in which they were grouped.

Standardized thematic area	Research topic
Agribusiness	Economic- Importation, Social, Marketing, Consumer preferences and acceptance, Profit analysis, Market analysis- Socio demographic
Genetics and Biotechnology	Genetic, Germplasm, Stable isotopes, Micropropagation tissue culture, Breeding
Ecosystem	Pollinators, Environmental impact, Greenhouse gas emissions
Soil and water management	Irrigation, Water stress, Soil characteristics
Plant Physiology	Abiotic stress, Ecophysiology, Phenology, Physiology, Reproductive (fruit setting, flowering, pollination, reproductive biology).
Crop management	Mulching, Fertilization, Shading nets, Rootstock, Planting, Compost, Modelling
Products	Processed fruits and by-products
Ethnobotany	Medical usage and plant use in social and cultural context
Fruit Quality	Food safety, Chemical composition, Physiochemical compositions, Maturity index, Nutraceutical, Quality index, Phenolic compounds, Postharvest technologies and agents
Pest and Pathology	Fungi, Mites, Insects, Arthropods, Virus, Bacteria

Studies conducted on several fruits or in more than one country were considered and categorized as “multiple”. A short outline was made for each of the eligible publications. To assess the expansion of tropical fruit cultivation, FAOSTAT data were used to calculate the percentage increase for each Mediterranean country over the past 20 years, followed by the computation of a simple (unweighted) average across countries. All descriptive and quantitative analysis were done using RStudio using the following packages (ggplot2, dplyr, ggalluvial) (4.3.3) (R Core Team, 2021).

## 3 Results

### 3.1 Publication trend over the years and research type

**Figure 1:**
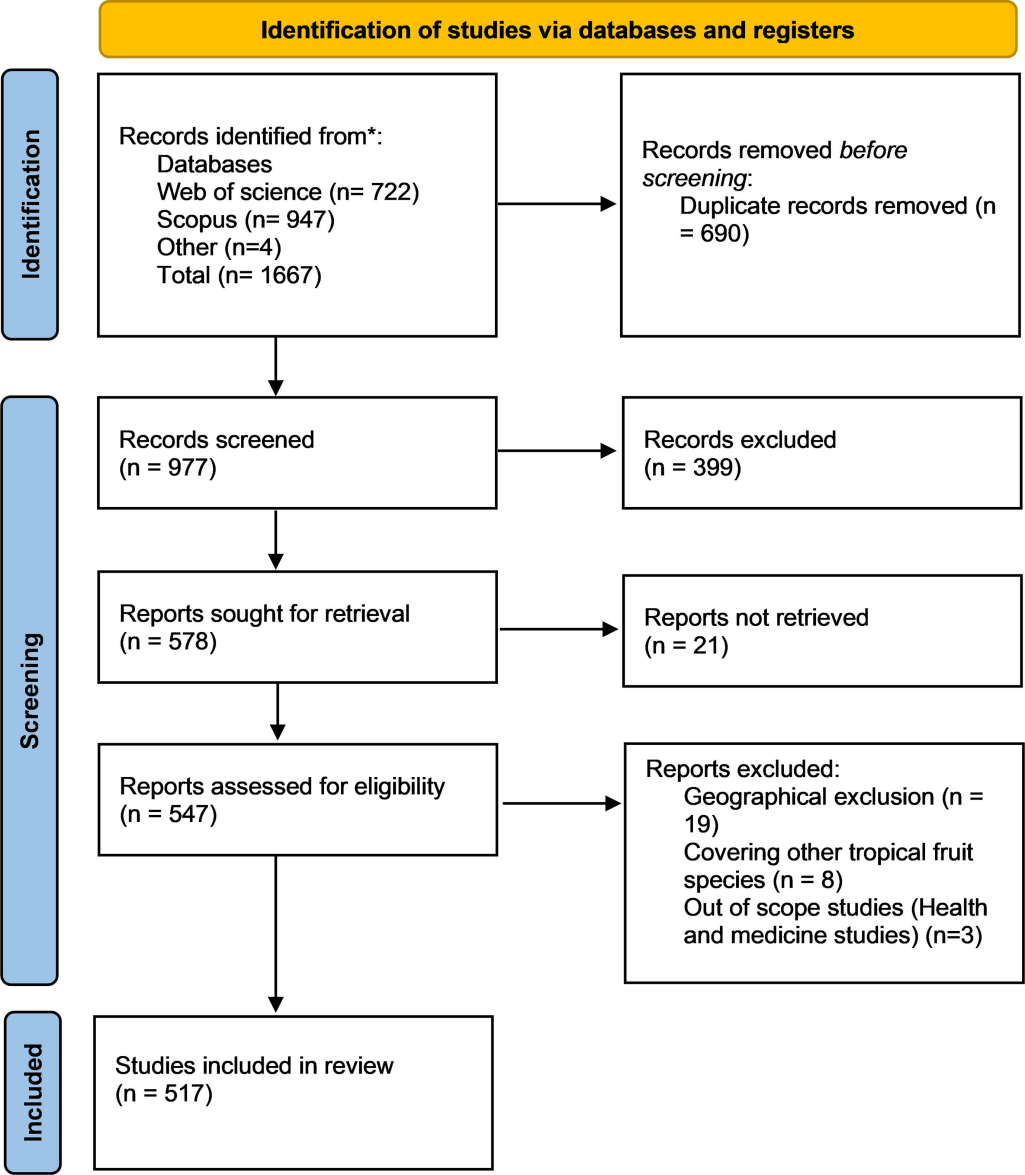
PRISMA flowchart for selecting studies, displaying the steps taken and the number of included and excluded article.

**Figure 2:**
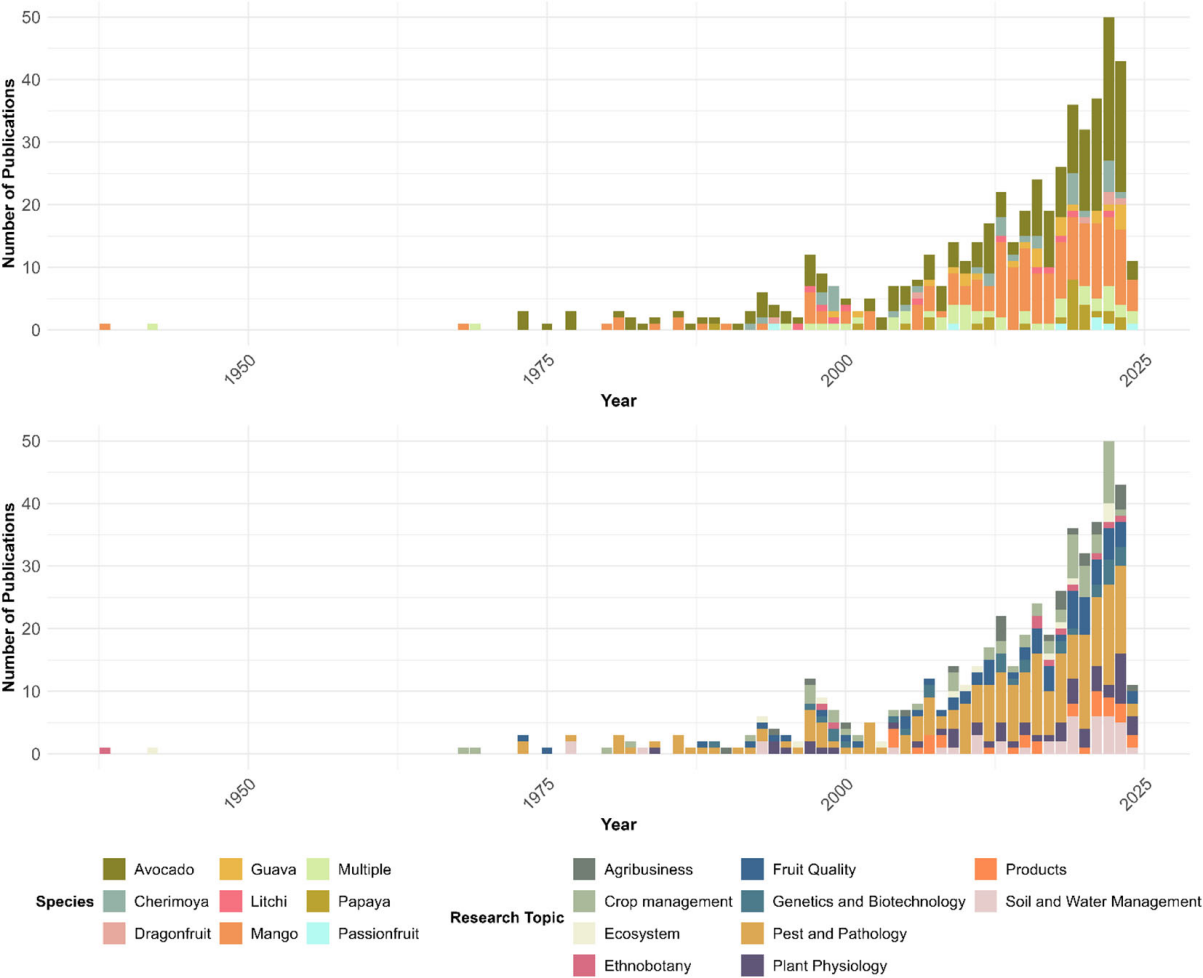
Stacked bar charts showing the temporal distribution of publications by species (top panel) and by research topic (bottom panel). The X-axis represents publication years, and the Y-axis indicates the number of publications.

Out of the 1667 publications initially obtained, a final dataset of 517 publications was retained (**Figure 1**). Most of the publications were published in the English language (96%). Few publications were written in Spanish, French, Turkish and Italian. The earliest publication dates back to 1938. “Over the past 90 years, an average of approximately five publications per year was recorded, increasing to 17 publications per year in the last decade (**Figure 2**). The highest number of publications was recorded in 2022 (n=50), followed by 2023 (n=43) and 2021 (n=37).

**Figure 3:**
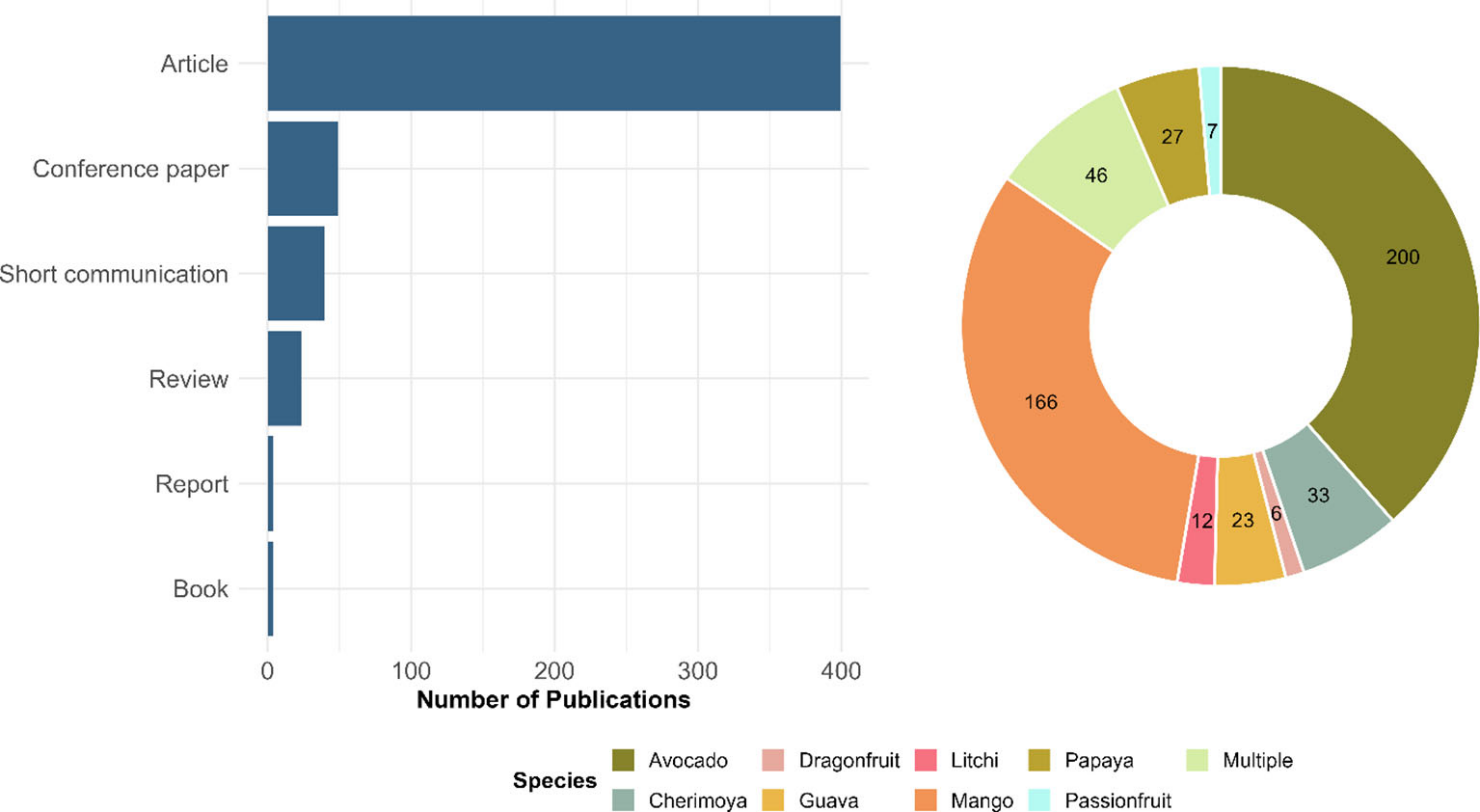
Number of publications per type of paper (left) and per species (right).

**Figure 4:**
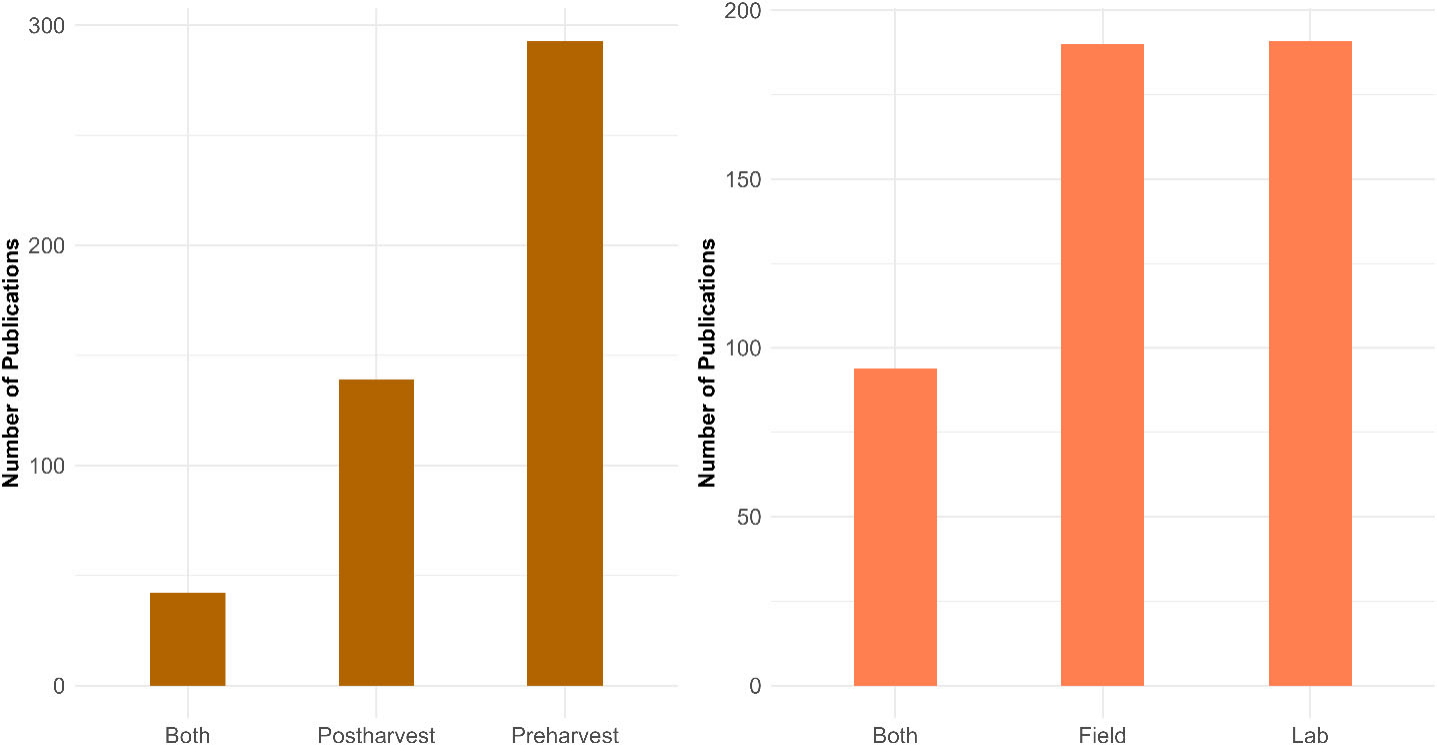
Number of publications per period of fruit life (postharvest/preharvest) (left) and per study area (field/laboratory) (right).

A total of 398 out of 517 publications were research articles (77%) (**Figure 3**). Conference papers accounted for 50 publications (9.6%), followed by short communications (n=39; 7.6%), reviews (n=21; 4%), and books and reports (n=4 each; <1%). “Most of the studies focused on avocado (n = 200; 38.7%) and mango (n = 166; 32.1%), followed by cherimoya (n = 33; 6.4%), papaya (n = 27; 5.2%), and guava (n = 23; 4.4%). Only a small proportion of publications focused on litchi, dragon fruit, and passion fruit. A notable share of the studies (n = 46; 8.9%) addressed multiple species (**Figure 3**). An approximately equal distribution was observed between laboratory (n = 188; 36.4%) and field studies (n = 183; 35.4%). In contrast, most research focused on the preharvest stage (n = 294; 56.9%), compared with the postharvest stage (n = 139; 26.9%) (**Figure 4**).

### 3.2 Geographical distribution of studied species

**Figure 5:**
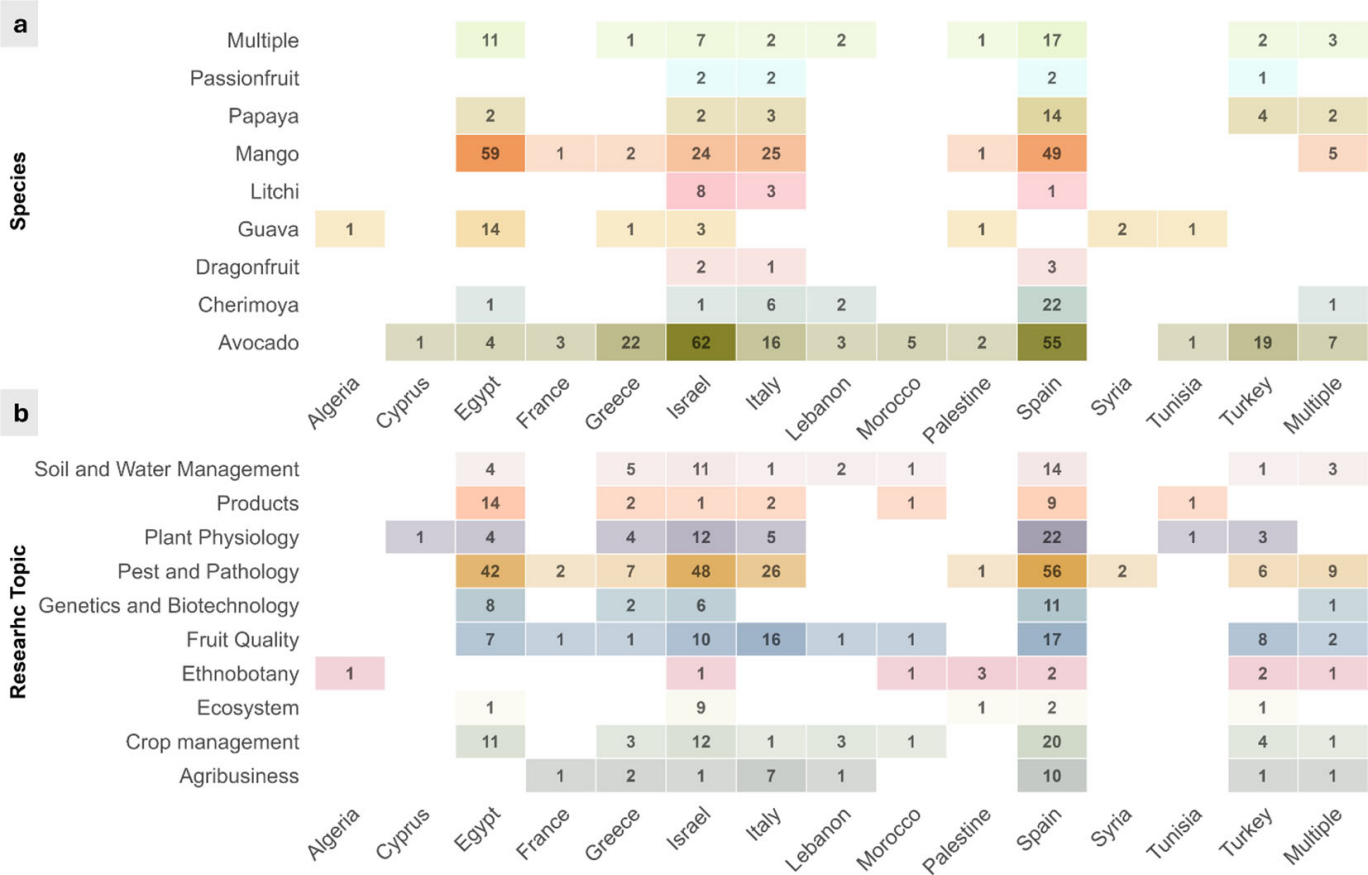
Heatmap representation of the number of publications by country and studied species **(a)**, and by country and research topic **(b)**; Tile colour intensity reflects the number of publications, with darker colours indicating higher values, while the exact number of publications is displayed within each cell.

An uneven distribution of research efforts was observed both between and within continents. Most studies were conducted in the European, followed by Asian and African part of the Mediterranean (**Figure 5a**). In Europe, Spain had the highest number of publications, covering all fruit types, with a strong focus on avocado and mango. Spain also led in research on cherimoya and papaya. Italy made a significant contribution as well, particularly on mango and avocado. No research on guava was conducted in European countries, except in Greece (n=1). Where most guava-related research was conducted in African countries, particularly in Egypt (n = 13), and to a lesser extent in Algeria and Tunisia (n = 1 each), as well as in Asian countries, including Israel (n = 3) and Palestine (n = 2).

In Asia, Israel stood out with over 100 publications, accounting for 13% of the total, primarily focusing on avocado and mango, followed by Turkey (n = 26), Lebanon (n = 7), and Palestine (n = 5). In Africa, Egypt dominated with 91 publications, mostly concerning mango. Other African countries had fewer contributions: Morocco (n = 5), Tunisia (n = 2), and Algeria (n = 1). Interestingly, a significant portion of the research was conducted either on multiple species or across multiple countries (n = 18) (**Figure 5a**).

### 3.3 Geographical distribution of research topic

The distribution of research thematic areas across countries revealed a degree of research specialization. Among all countries, only Spain and Israel addressed all the thematic areas (**Figure 5b****).** Research on “Pest and Pathology” and “Crop management” was particularly prominent in Spain (n=56 and n=20, respectively), Egypt (n=42 and n=11), and Israel (n=48 and n=12). Studies on fruit ‘Products’, especially those focusing on fruit by-products, were published in seven countries: most of them came from Egypt and Spain, while France, Italy, Tunisia, Israel, and Morocco had only one or two publications on the topic. “Plant physiology” was primarily investigated by researchers from Spain and Israel. It was also the focus of a single study conducted in Cyprus. Research on “Genetics and Biotechnology” was mainly carried out in Egypt, Spain, and Israel, with only two studies conducted in Greece. Also, studies regarding ‘Soil and Water Management’ were conducted mostly in Spain and Israel. ‘Ethnobotany’ research was more commonly investigated in North African countries such as Algeria and Morocco, as well as in Middle Eastern countries including Palestine (n=3), Turkey (n=2) and Israel (n=1). A total of 14 studies focused on ‘Ecosystem’, of these, nine were published in Israel, two in Spain, and one each in Egypt, Turkey, and Palestine. Finally, research on “Agribusiness” and consumer preferences was mainly published in Spain (n=10) and Italy (n=7), followed by Greece (n=2). Single publications on this theme were also found in France, Israel, Lebanon, and Turkey (**Figure 5b**).

### 3.4 Combination of research topic and fruit species

Across all species, ‘Pest and Pathology’ research topic emerged as the most frequently studied topic, particularly within mango (46.99% of mango-related studies), avocado (42.5% of avocado-related studies), and guava (43.48% of guava-related studies). ‘Fruit Quality’ was major research topic, particularly in cherimoya (27.27%), papaya (22.22%), guava (21.74%), and litchi (25.0%). ‘Genetics and Biotechnology’ research were also well-represented in cherimoya (21.21%), guava (13.04%), and litchi (8.33%), while ‘Plant Physiology’ studies accounted for 50% of studies of dragon fruit, 12.2% of cherimoya, and 14.81% papaya publications.

**Figure 6:**
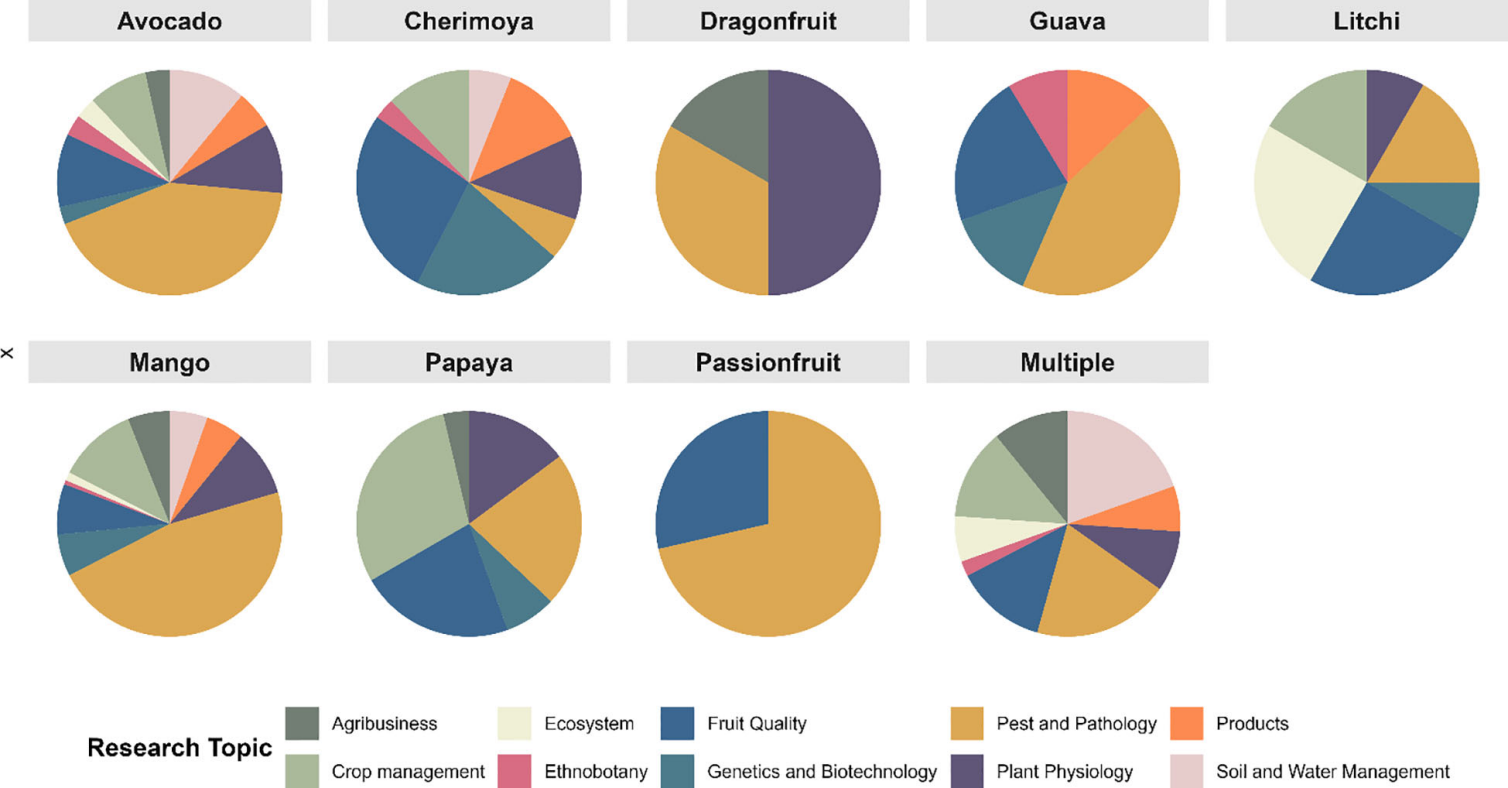
Percentage of research topic studied per each species.

In terms of “Crop management” research topic, notable proportions were found in papaya (29.63%), cherimoya (12.12%), and mango (11.45%). ‘Soil and Water Management’ topic was most studied topic in avocado publications (11.0%). Less commonly addressed topics included “Ethnobotany”, “Ecosystem” and “Agribusiness” studies. For instance, ecosystem-related studies appeared mainly in litchi publications (25.0%) and multiple fruits (6.38%), while “Agribusiness” topic was addressed primarily under multiple fruits (10.64%) and mango (6.02%) (**Figure 6**).

### 3.5 Studied plant parts

**Figure 7:**
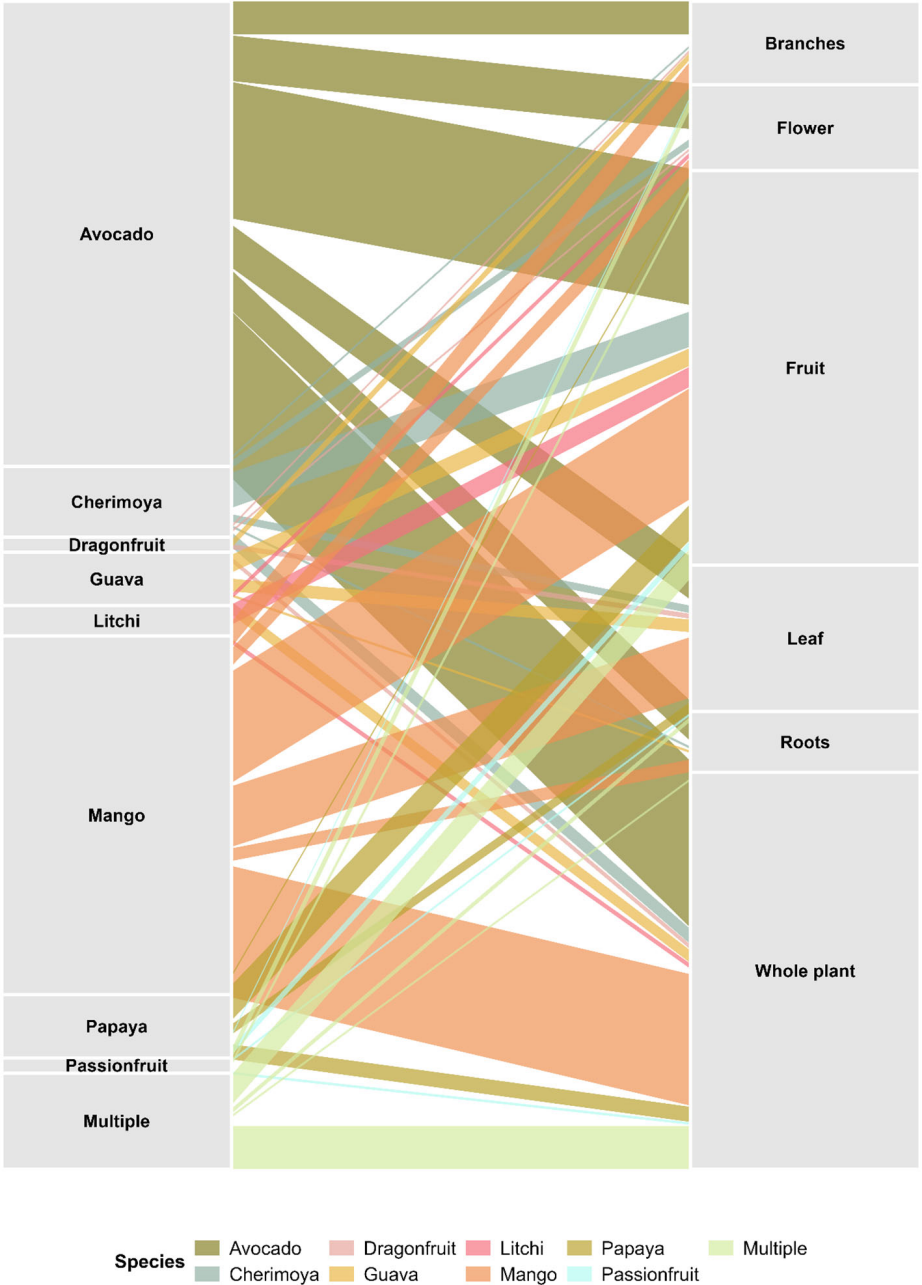
Relations between part of plant studied and species.

For each publication, the plant part examined in the study was identified. Overall, more than half of the studies focused either on the whole plant or specifically on the fruit, with an equal number of publications (n = 155). These were followed by studies focusing on the leaf (n = 56), flowers (n = 34), branches (n = 27), and roots (n = 24). In the case of avocado, most studies focused on the whole plant, fruit, flower, and branches, with fewer studies examining the leaf and roots. For dragon fruit, most studies focused on the cladode, which is classified here as a leaf. In contrast, few studies were dedicated specifically to the flower, with most publications addressing the whole plant, fruit, or leaf. Guava publications mainly investigated the fruit and the leaf. Approximately 70% of studies on litchi focused on fruit. Research on papaya primarily examined the fruit and the whole plant, with fewer studies focusing on the leaf and flower. Cherimoya was mainly studied for its fruit and leaves, while studies on passionfruit were distributed almost equally between the fruit and whole plant, and only one publication was focusing on the leaf (**Figure 7**).

## 4 Discussion

Following the quantitative analysis of the scientific literature on tropical fruit species in Mediterranean countries, a qualitative synthesis was conducted to identify key research trends, thematic imbalances, and knowledge gaps across species. Overall, research efforts are unevenly distributed, with a strong concentration on economically important crops such as mango and avocado, while other species remain comparatively understudied. This bias appears to be largely driven by the economic importance and production scale of these crops. The increase in publications over time reflects both their growing relevance and their expansion across the Mediterranean region, as well as the constraints associated with their adaptation to local environmental conditions. These constraints are particularly evident in the predominance of studies focused on pest and pathology and crop management, indicating a challenging problem-driven research approach. In contrast, areas such as plant physiology, climate adaptation, and long-term sustainability remain comparatively less investigated, although interest in these topics has increased in recent decades. Furthermore, a geographical bias in research output is evident, influenced not only by production levels but also by disparities in research funding, infrastructure, and institutional capacity across countries. While some Mediterranean countries contribute significantly to tropical fruit production, their scientific output remains relatively limited, likely due to constraints in research resources and access to indexed publication platforms. This disparity is particularly evident among geographically close countries that exhibit markedly different publication outputs, such as Lebanon and Israel, or Syria and Turkey. These differences may also reflect variations in national research capacity, funding availability, and broader socio-economic conditions that influence research development and priorities. When compared to the global context, both in terms of scientific production and economic importance, similar trends and patterns are observed. However, the Mediterranean Basin still represents only a small share of this production. Despite this limited contribution, the sector is expanding and shows promising potential for future development. The most relevant highlights for each species found across the examined publications are presented below.

### 4.1 Mango

Among the different tropical fruits, mango is considered as the pioneer and early introduced tropical fruit in the Mediterranean. In fact, the first written scientific report on its cultivation across the Mediterranean dates to 1938 mentioning its acclimatization in Palestine. It introduced as a potential crop to bridge the local seasonal gap between grape and citrus seasons (Oppenheimer, 1938). Gradually mango has evolved from an exotic to a key crop in several countries around the Mediterranean. In Europe, mango fruit demand has grown steadily in recent years. However local production remains insufficient to meet this demand (Liguori et al., 2018), and imports from non-European Mediterranean countries frequently fail to comply with EU pesticide residue standards (Soliman et al., 2017; EFSA, (European Food Safety Authority) et al., 2021), and may promote alien pest introduction (Steffen et al., 2015).

Many international mango varieties are cultivated across the Mediterranean, and local varieties. Across local germplasm, a significant morphological variation and high levels of polymorphism were observed in the Egyptian mango germplasm. Through RAPD markers, Mansour et al. (2014) revealed 78% polymorphism. The Israeli mango collection, analysed with 293 (SNPs), was divided into two groups: accessions from Southeast Asia and India, and accessions from Florida and Israel, the latter being more polymorphic. Similarly, Aseeri et al. (2021) used fruit quality characteristics and ISSR markers, demonstrating that the new strains of cv. Sukari had good commercial potential. ISSR with (SCoT) markers revealed a polymorphism of 53.84% on 23 mango genotypes from El-Giza and highlighted new cultivars such as Aya, Kasturi, Maya and Omer as promising for future breeding programmes (El-Shenawy Ghounim et al., 2022). Through stable isotopes (δ15N, δ18O, δ34S) were identified as key indicators for distinguishing Spanish mangoes from those originating from foreign markets (Muñoz-Redondo et al., 2021). The results of the studies underline how the presence of germplasm collections is fundamental for preserving international and local accessions and serves as a vault of useful genetic materials.

Aside from cultivar and varietal selection, rootstock choice is also fundamental for the adaptation of mango (Oppenheimer, 1968; Homsky, 1996; Durán Zuazo et al., 2006; Galán Saúco, 2019). Sabre, 14–6 and 14–7 are considered suitable rootstocks for Mango in a hot arid climate, while Gomera 3 is more adaptable in areas with milder summers and higher water availability. However, mango varieties cultivated in the Mediterranean basin face varying degrees of limitations to their cultivation, primarily due to environmental conditions. Several abiotic and biotic factors have been found to be detrimental, including low temperature and heavy winds (Comino et al., 2014), water stress (Fonollá et al., 2023) and water quality issues (Helaly et al., 2018).

To overcome environmental limitations, mango is widely grown under protected cultivations system in several regions. Many studies were published on the performance of this species when cultivated inside of greenhouses or other forms of protected cultivation (Lionakis and Loxou, 1996; Medina et al., 2009; Galán Saúco, 2015). Some drawbacks were pointed out such as the necessity of avoiding flowering in the young trees, excessive heat and pollination deficits. A study conducted in Sicily on mango evidenced good performances in terms of photosynthesis under greenhouse conditions despite the high temperatures (50 °C) (Gugliuzza et al., 2023). Several pollinators, including hoverflies (*E. aeneus*), were tested and led to high yields in Osteen mango under greenhouse conditions in Spain (Sánchez et al., 2022). Besides greenhouses cultivation, shading and windbreaking nets emerged lately as viable alternatives (Scuderi et al., 2022). Other protective measures, including windbreaks, overhead sprinklers, and gypsum or kaolin applications, help trees withstand extreme heat, low humidity, and sunburn (Bellitti et al., 2025).

Another key factor influencing tree resilience is irrigation. Mediterranean subtropical environments are characterized by limited and fluctuating water resources, high evaporative demand, and recurrent droughts, which strongly affect orchard management and productivity. Sustained-deficit irrigation (SDI) strategies have been widely investigated in the subtropical climate of the coast of Spain. Durán Zuazo et al. (2011) reported that applying 50% ETc (2,580 m³ ha^-^¹) provided the highest mango yields and water-use efficiency (7.1 kg m^-^³). A recent study in the same region (Durán Zuazo et al., 2021) showed that water stress reduces fruit size and yield, with 33% ETc producing smaller fruits, though with significantly improved fruit quality. Similarly, Levin et al. (2018) demonstrated that mango trees are highly sensitive to water deficit during the final fruit growth and post-harvest stages, highlighting the critical role of phenological stage–based irrigation management to balance yield and water savings. Alhashimi et al. (2023) showed that partial rootzone drying with three layers of organic mulch-maintained yields comparable to full irrigation (~10–13 t ha^-^¹) while halving water use and boosting water productivity to 5.6 kg m^-^³, with notable improvements in fruit quality. Durán Zuazo et al. (2019) provided reference Kc values for Mediterranean terraces (Kc ≈ 0.43 flowering, 0.67 fruit set, 0.63 fruit growth) and annual irrigation needs of 4,166–5,326 m³ ha^-^¹; under full irrigation, yield averaged 24.1 kg tree^-^¹ and WUE reached 3.1 kg m^-^³. Beyond orchard-level management, broader sustainability issues emerge. At landscape scale in SE Spain, Durán Zuazo et al. (2013) linked subtropical crop expansion (including mango) to erosion, aquifer depletion, salinization risk and leaching risks (Rodríguez Pleguezuelo et al., 2011). Finally, as for the water-energy-carbon nexus, Tocados-Franco et al. (2024) projected a 30% rise in water demand by 2030 in Spain, resulting in a 42 hm³ deficit. Few works on mango fertilization were also reported where boron fertilization associated with nitrogen supply alleviated the phenomenon of alternate bearing (El-Motaium et al., 2019; Shaban et al., 2019; El-Hoseiny et al., 2020).

On the hand, the increase in the international trade of plants has facilitated the introduction of several exotic pest species (Cano, 2015; Boyero et al., 2017). Scale insects are generally considered to have high potential as invasive alien species, causing direct and indirect damage (García Morales et al., 2016). *Aulacaspis tubercularis* Newstead (Hemiptera: Diaspididae), known as White Mango Scale (WMS), is a highly polyphagous invasive species, a potential quarantine pest in the European Union (Lo Verde et al., 2020; Bragard et al., 2022). After its first record in Egypt and Israel (Balachowsky, 1954), it was found in Italy (Porcelli, 1990) and Spain (Del Pino et al., 2021b). In areas where it has stabilized, it reaches from two (Bragard et al., 2022) to four (Mokhtar et al., 2015; Salem et al., 2015) population peaks. Among natural enemies, the parasitoids, *Encarsia* spp. and *Encarsia citrina* Craw parasitized both males and females of WMS (Del Pino et al., 2023). The presence of several WMS parasitoids (Abd-Rabou, 2014) and predators (Abo-Shanab, 2012; Sayed, 2012) was recorded in Egypt. Yet, the natural occurrence is still insufficient to keep populations below economic thresholds, and additional control measures are regularly required (Labuschagne et al., 1995; Daneel and Joubert, 2006). Several integrated control strategies can be employed. An effective physical method was represented by fruit bagging, which reduced WMS fruit damage by 68.09%. Paraffin oil (1.25%) and botanical mixtures were also found effective in reducing the instars of WMS (Siam and Othman, 2020; Del Pino et al., 2021a).

Other scale insects species are present on mangoes across the basin: *Milviscutulus mangiferae* Green (Hemiptera: Coccidae), known as Mango Shield Scale (MSS) is a potential EU quarantine pest (EFSA et al., 2023a). It was found in Israel as a pest of economic importance (Kfir and Davis, 1981; Wysoki et al., 1992), in Italy (Pellizzari and Porcelli, 2014) and Egypt (Abd-Rabou and Evans, 2018). For *Kilifia acuminata* Signoret (Hemiptera: Coccidae), economic threshold and injury levels were set in Egypt (Nabil, 2013; Mokhtar et al., 2015). *Icerya seychellarum* Westwood (Hemiptera: Coccomorpha: Monophlebidae), was recently observed in Italy (Lo Verde et al., 2020), after being reported 50 years earlier in Egypt (Ezz and Samhan, 1969). Other scale insects species are present on mangoes across the basin: *Milviscutulus mangiferae* Green (Hemiptera: Coccidae), known as Mango Shield Scale (MSS) is a potential EU quarantine pest (EFSA et al., 2023a). It was found in Israel as a pest of economic importance (Kfir and David, 1981; Wysoki et al., 1992), in Italy (Pellizzari and Porcelli, 2014) and Egypt (Abd-Rabou and Evans, 2018). For *Kilifia acuminata* Signoret (Hemiptera: Coccidae), economic threshold and injury levels were set in Egypt (Nabil, 2013; Mokhtar et al., 2015). *Icerya seychellarum* Westwood (Hemiptera: Coccomorpha: Monophlebidae), was recently observed in Italy (Lo Verde et al., 2020), after being reported 50 years earlier in Egypt (Ezz and Samhan, 1969).

As for Diptera Tephritidae, *Bactrocera zonata* Saunders and *Ceratitis capitata* Wiedemann are among the most damaging in mango (Wysoki et al., 1992; El-Gendy, 2017). Two major thrips*, Scirtothrips mangiferae* Priesner and *Frankliniella occidentalis* Pergande (*Thysanoptera*: *Thripidae*) are found on young mango leaves and considered economically harmful.

Mites are another hindering factor. *Oligonychus mangiferus* Rahman and Sapra (Acarina: Tetranychidae) (Gerson, 1992), *Aceria mangiferae* Sayed (Acarina: Eriophyidae), *Cisaberoptus kenyae* Keifer (Acarina: *Eriophyidae*) (Wysoki et al., 1992), *Metaculus mangiferae* Attiah (Acarina: *Eriophyidae*) were observed in Egypt and Israel (Elhalawany et al., 2023). Recently, *Cisaberoptus kenyae* Keifer was also reported for the first time in Europe in Sicily (Giordano et al., 2025). Predation tests in the laboratory have shown that *Amblyseius swirskii* Athias-Henriot is a primary predator of these species (Abou-Awad et al., 2010).

Powdery mildew, caused by *Oidium mangiferae* Berthet, recently renamed as *Pseudoidium anacardii* (F. Noack) (Braun, 2012), provokes damage on inflorescences and fruit set, compromising crop yield. Sulphur, mineral oils, acetic acid application on affected plants showed great control for this fungus (Nofal and Haggag, 2006; Reuveni et al., 2018; El-Mslmany et al., 2020). *Fusarium mangiferae*, became a major problem in Egypt in 1958 (Ibrahim et al., 1975), with losses up to $35 million/year (Ploetz et al., 2002). In 2006 it was first reported in Spain on different mango varieties (Crespo et al., 2012). Both conidial germination and colony growth require temperatures >5 °C and reached a peak at 28 and 25 °C, respectively (Gamliel-Atinsky et al., 2009).

Ismail et al. (2013) identified *Neofusicoccum parvum* and *N. australe* as the agents responsible for mango dieback. *N. parvum* was mainly observed on young mango plants (Arjona-Girona and López-Herrera, 2016), and on nursery (Polizzi et al., 2023). *Verticillium dahliae*, the agent of *Verticillium* wilt, also causes dieback of infected mango branches (Baeza-Montañez et al., 2010; Youssef et al., 2014). Potassium sorbate, ammonium bicarbonate and disodium EDTA showed the best performance as alternative control methods. To note, dieback can also be caused by *Nigrospora sphaerica*, *Lasiodiplodia theobromae* and *Neoscytalidium dimidiatum* (Ismail et al., 2012; Youssef et al., 2022; Sharma et al., 2024).

In the Mediterranean basin, the main agents responsible for mango anthracnose belong to the genus *Colletotrichum* (Ismail et al., 2015; Ismail and El-Ganainy, 2022). The extract of *Plantago sinaica* inhibited *in vitro* the growth of *C. gloeosporioides* mycelium (Baka and Mousa, 2020). When anthracnose is effectively controlled, stem-end rot (SER) can be the primary cause of post-harvest losses in mangoes. In Israel, the main causative agents responsible for the disease are *Alternaria alternata*, *C. gloeosporioides*, *Lasiodiplodia theobromae* (Diskin et al., 2019).

*Pseudomonas syringae* pv. *syringae* is the causal agent of Bacterial Apical Necrosis (BAN) in mango trees. It was initially detected in Spain on several cultivars (Cazorla et al., 1998), and more recently reported in Greece (Trantas et al., 2017). The various symptoms of the disease are indicative of high heterogeneity of the pathovars of the bacterium and close coevolution with the host, resulting in phylogenetic subgroups (PSGs) (Gutiérrez-Barranquero et al., 2013, 2019, 2022; Aiello et al., 2015). Bordeaux mixture (BM) is generally used, but it does not act as a bactericide. According to (Cazorla et al., 2006), alternative treatments are acibenzolar-S-methyl and the phosphonate derivative fosetyl-Al. A treatment compatible with organic farming, is silicone gel, which has shown similar efficacy to conventional BM treatment (Gutiérrez-Barranquero et al., 2012).

The increasing cultivation and valorisation of mango in Mediterranean countries has prompted numerous studies aimed at improving its postharvest quality, extending its shelf life, and exploiting its by-products. In Sicily, locally grown mangoes have shown quality equal or superior to imported tropical fruits, especially when harvested at full maturity (Liguori et al., 2018; Gentile et al., 2019; Farina et al., 2020a). Non-destructive techniques such as VIS-NIR and TRS spectroscopy have proven to be reliable tools for monitoring ripening and internal quality (Cortés et al., 2016; Vanoli et al., 2016). Yet postharvest management remain a critical point in maintaining fruit shelf-life and quality. The use of natural edible coatings (Feygenberg et al., 2005) and bioactive compounds like melatonin, tragacanth gum or neem oil (Passafiume et al., 2022; Khedr and Al-Khayri, 2023) helped preserve sensory and nutritional properties during cold storage.

Beyond the fruit itself, mango seeds have long been considered a valuable by-product. Several studies have analysed their amino acid, phenolic, and lipid profiles, highlighting their value as functional ingredients for food and industrial applications (Abdalla et al., 2007b; Alañón et al., 2021; Mustafa et al., 2024). Mango seed extracts have proven to be powerful antioxidants and antimicrobials, effective in improving the oxidative stability of foods and extending the shelf life of dairy and snack products (Abdalla et al., 2007a). Furthermore, seed extracts have also been effectively used as natural coagulants for drinking water treatment in rural areas (Elmolla et al., 2020). Other works have analysed the influence of extraction methods and variety on the phenolic composition of mango by-products (Dorta et al., 2014) and characterized the metabolites of kernels from different Egyptian regions (El-Shabasy et al., 2024), paving new ways for nutraceutical and industrial exploitation (Chulibert et al., 2024).

Regarding processing, the quality of dried mango slices was assessed for Keitt and Osteen cultivars, with the latter showing greater sensitivity to volatile loss at high drying temperatures, negatively affecting sensory quality (Russo et al., 2019). On the food safety front, the development of multiresidue methods for the rapid analysis of pesticides in mangoes enabled reliable detection of residues below maximum residue limits (MRLs), allowing to establish a monitoring protocol of tropical fruits (Martnez Vidal et al., 2007). Lastly, mango leaf extracts showed remarkable antimicrobial activity against *S. aureus* and *E. coli*, due to bioactive compounds such as trans-caryophyllene and α-humulene suggesting its potential use as a natural food preservative (Ouf et al., 2021). Indeed, mango cultivation represents both the opportunities and the challenges of introducing tropical crops into Mediterranean systems. Additional multidisciplinary research is required to fully realize its potential and ensure long-term sustainable cultivation. Overall, the literature on mango is highly comprehensive, reflecting its economic importance and long history of introduction in the Mediterranean region. However, research is strongly skewed toward pest and disease management and irrigation strategies, while comparatively less attention has been given to long-term climate resilience and integrated sustainability approaches. This highlights the need for more multidisciplinary studies addressing future climatic challenges.

### 4.2 Avocado

Avocado is highly regarded for the fruit’s nutritional value and pleasant taste, but also for its medicinal properties in various folks and cultures. This is evident across the Mediterranean basin: in Turkey, leaves are widely used as a traditional remedy for kidney-related issues (Erdem et al., 2016; Köroğlu and Kendir, 2018). Avocado honey has also demonstrated great anti-amyloidogenic capacity (Romero-Márquez et al., 2023), and is therefore used in many Mediterranean countries to treat dermatological diseases and skin inflammation (Shawahna and Jaradat, 2017; Ajjoun et al., 2022).

Interestingly, the literature search revealed the fundamental contribution of Mediterranean researchers to the basic knowledge of avocado genetics. Almost 500 different avocado cultivars were described, according to their country of origin, race and flowering group (Lahav and Gazit, 1994). Genetic diversity within avocado germplasm in the E.E. La Mayora collection (Spain) was studied using microsatellite markers, which allowed clear identification of all accessions except for some “Hass” mutations (Alcaraz and Hormaza, 2007). With the same aim, stable isotope and elemental profiles have been applied to trace the geographical origin of avocado from Spain and other producing countries, achieving 98% accuracy (Muñoz-Redondo et al., 2022).

In many areas of the Mediterranean basin, avocado faces abiotic and biotic conditions that may be detrimental to plant and fruit health (Lomas, 1992). Avoiding heat stress and sunburn damage was one of the main interests of many studies. Different mitigation approaches showed promising results, including over-canopy irrigation (Lazare et al., 2022b) and shading nets (Rubinovich et al., 2023). On the other hand, cold temperatures can also cause just as serious damage as excessive heat: in this context, the use of a wind machine helped reduce frost damage on avocado varieties (Bar-Noy et al., 2019).

In semi-arid regions of the Mediterranean basin water scarcity, salinity, and soil constraints represent major challenges for sustainable avocado production, and many studies have focused on optimizing irrigation and improving water-use efficiency. A seminal study on three commercial varieties showed that ~ 1200 mm of yearly rainfall can be enough for avocado on clay soils (Lahav and Kalmar, 1983). Early foundational studies highlighted the importance of irrigation frequency, with Lahav and Kalmar (1977) showing that avocado trees extracted most water from the upper 60 cm of soil and that irrigation amounts of 6680 m³ ha^-^¹ year^-^¹, offered the best balance between water efficiency, growth control, and yield consistency. Research in Crete (Michelakis et al., 1993) demonstrated that drip irrigation at 0.6 of Class A pan evaporation improved root development and yield, while in Morocco, Taleb et al. (2022) found that replacing drip irrigation with micro-sprinklers improved root activity, reduced irrigation amounts by over 300,000 l/ha and increased yield by 33% (Moreno-Ortega et al., 2019). in southern Spain observed that irrigation at 100% of ETc maximized yields at 23.2 t ha^-^¹, while moderate deficit at 60% ETc reduced yield by about 30% yet improved water productivity and reduced alternate bearing, while severe deficit at 40% ETc lowered fruit size but maintained acceptable yields. Similarly, Durán Zuazo et al. (2021) identified 75% ETc as the optimal compromise between productivity, water savings, and fruit quality. Salinity has emerged as a critical limiting factor for avocado, as yields decline when irrigation water exceeds 0.75 dS m^-^¹ EC. Salt-tolerant rootstocks such as ‘Dusa’ and ‘Nabal’ show promising results (Kourgialas and Dokou, 2021). Irrigation with treated wastewater led to many plant health issues and is therefore not recommended (Nadav et al., 2013; Yalin et al., 2017, 2021). Research on rootstocks and fertigation strategies has further refined irrigation management: (Cohen et al., 2023) found that seedling rootstocks are more productive than clonal West Indian rootstocks when both are irrigated with recycled water (Winer, 2022). Advanced water status monitoring instruments, such as dendrometers, fruit growth sensors or digital tensiometers and mass balance modelling allowed to optimize irrigation scheduling (Karam et al., 2019). Kourgialas et al. (2022) developed a GIS-based platform that integrates meteorological, satellite and soil analyses data to deliver plot-level irrigation recommendations in Crete. As for the sustainability of avocado cultivation, aquifer overexploitation, soil erosion, and risk of salinization from marine intrusion was documented in south-eastern Spain (Durán Zuazo et al., 2013). Soil fertility studies further showed that avocado residues decompose slowly and immobilize nitrogen compared to faster-releasing species like cherimoya (Rodríguez Pleguezuelo et al., 2009), while sustainable soil-improving practices in avocado orchards reduced erosion by 34% in Crete and increased soil organic carbon and hydraulic conductivity (Tsanis et al., 2021). Collectively, these findings demonstrate that avocado production in Mediterranean and arid regions is highly sensitive to irrigation frequency, water quality, and soil management. While optimized irrigation schedules and deficit strategies can improve water-use efficiency and fruit quality, threats from salinity, wastewater use, and unsustainable expansion persist. Technological tools such as phyto-monitoring, tensiometry, and GIS platforms offer promising advances, but long-term sustainability will ultimately depend on integrating these innovations with soil conservation, and policies that address climate change, water scarcity, and energy costs. To further enhance sustainability of this species’ cultivation, it is recommended to improve irrigation systems and reduce the use of chemical fertilizers.

Studies on fertilization of avocado have covered foliar application of micronutrients (Lazare et al., 2022a) or, more commonly, crop management techniques aimed at improving soil quality and increasing fruit yield (Bonilla et al., 2012; Aguirre-Arcos et al., 2022; Tzatzani et al., 2022). Avocado trees, in fact, have a low fruit set percentage due to pollen and pollination problems. Alcaraz and Hormaza in Spain evidenced that the percentage of flowers with pollen on the stigma varies along the flowering season, probably due to changes in temperature that affect not only the floral behaviour but also pollinator activity. Thus, natural pollination conditions might not be enough to ensure a good yield. Honeybees may not be the most efficient pollinators for avocado, as even with a substantial number of hives, the percentage of flowers receiving pollen during the female stage remains very low. Therefore, enhancing pollinator diversity and increasing their numbers could be a viable strategy to improve the percentage of avocado flowers receiving pollen during the female flowering stage (Boldingh et al., 2016; Alcaraz and Hormaza, 2021, 2024).

With the increasing cultivation of avocado in Mediterranean countries, many studies have turned their attention on pest and pathogens impacting fruit yield and quality. As part of different control strategies, many studies proposed biological control methods. For instance, the suppressive capacity of various organic amendments or soil application of a formulated biocontrol rhizobacterium *Pseudomonas chlororaphis* PCL1606, as well as soil solarization that have been demonstrated to be effective against white root rot caused by the fungus *Rosellinia necatrix* (Tienda et al., 2020; Bonilla et al., 2015; López-Herrera et al., 1998). In 2021, a first finding of *R. necatrix* on avocado in Italy, was reported in new areas of cultivation (Fiorenza et al., 2021). In recent years, some authors have conducted field experiments using attractant traps based on quercivorol and other repellent substances to develop innovative “push-pull” control methods. Promising results have been obtained in the monitoring and mass trapping of the polyphagous shot hole borer (*Euwallacea fornicatus* Eichhoff), which infests avocado branches in Israel (Byers et al., 2017, 2020, 2021), particularly affecting the cultivar ‘Hass’ (Mendel et al., 2017). Among insects end mite, the long-tailed mealybug *Pseudococcus longispinus* (Targioni Tozzetti, 1867) (Hemiptera: Pseudococcidae) and Persea Mite *Oligonychus perseae* Tuttle, Baker and Abbatiello1976, (Acari: Tetranychidae) are considered a key pest of avocado (Wysoki et al., 1977; Zappala et al., 2015), the fungus *Neofusicoccum parvum* which is one of the causing agents of anthracnose (Guarnaccia et al., 2016), and the Avocado Sunblotch Viroid (ASBVd), which meets the EFSA’s criteria for designation as a potential quarantine pest for the EU (EFSA et al., 2023b). ASBVd was detected in Greece in 2016, where symptoms typical of avocado sunspot disease were observed, with greenish and yellowish-white depressed craters on the fruit (Lotos et al., 2018). Avocado is also affected by several rot diseases impacting both fruits and roots. In Spain and Italy, dark brown lesions and necrotic spots in the pulp have been observed on fruits (López-Herrera et al., 2005; Garibaldi et al., 2012). These rots are not limited to fruits but also affect the root system. Several pathogens have been identified as causal agents, including *Ilyonectria macrodidyma* (Vitale et al., 2012) and *Phytophthora cinnamomi* (López-Herrera and Pérez-Jiménez, 1995; Cacciola et al., 1998).

Harvesting day in avocado is critical and varies across regions; in this context, a study conducted in Lebanon (Merheb et al., 2020) aimed at establishing a maturity index for harvesting avocados at the ideal time, based on data collected in the Mediterranean climate. From a consumer perspective, alongside maturity index, fruit quality intended as flesh firmness, texture, and nutritional value is considered a major factor in consumer choice (Giuggioli et al., 2023). Understanding consumer preferences in terms of taste, variety, and other attributes is essential for both small-scale farmers and new producers helping them to meet dynamic market demands and needs (Migliore et al., 2018).

In this context several studies on the chemical and composition and nutraceutical value of avocado were conducted in many different countries across the Mediterranean basin. In Turkey, fruit’s boron concentrations averaged 29.2 ± 1.11 µg g^-1^ (Kuru et al., 2019). The lipid content of the avocado mesocarp varied widely depending on the variety, ranging from 1,628 to 11,116 mg per 100 g dry weight. The primary fatty acids identified include oleic acid (up to 31%), palmitic, palmitoleic, and linoleic acids. Studies on oils from four Moroccan avocado cultivars showed oleic acid as the predominant fatty acid (up to 65%), with the Fuerte variety containing significantly higher sterol levels compared to Ettinger (2,686.8 mg kg^-1^) (Nasri et al., 2021). In avocados of different cultivars grown in Egypt, Hass had the highest concentrations of carotenoids, total phenolics, and epicatechin (Rozan et al., 2021). In Greek, Zutano avocado oil, 44 volatile compounds were identified, with terpenes representing the dominant group (61.7%). The oil’s aroma intensity was observed to peak after four days of storage (Xagoraris et al., 2022). A Lebanese study evaluating eight avocado cultivars grown at altitudes ranging from 50 to 400 meters found that the Fuerte cultivar had the highest dry matter content (28.5%) and oil content (21.6%), whereas the Reed cultivar showed the lowest values (dry matter 8.2%, oil content 9.7%). A strong positive correlation between dry matter and oil content has also been reported (Salameh et al., 2022). Research indicates that avocado has strong potential as a sustainable and profitable crop in the Mediterranean, even under conditions of limited water availability, while being comparatively less affected by major pathological constraints. Similar to mango, avocado research is extensive and technologically advanced, particularly in irrigation and water-use efficiency. However, the strong emphasis on productivity and resource optimization contrasts with the relatively limited attention given to socio-economic sustainability and the long-term environmental impacts of crop expansion, especially in water-scarce regions. Research indicates that avocado has the potential to serve as a sustainable and profitable crop in the Mediterranean, even under restricted water availability, while being comparatively less affected by major pathological constraints.

### 4.3 Papaya

In the Mediterranean countries, papaya is almost exclusively cultivated under greenhouses, due to the high fragility of the plant (Gunes and Gübbük, 2012; Honoré et al., 2019). Most of the studies regarding “Crop management” of this species were conducted by a single research group in Spain and investigate the best practices to carry out inside greenhouses, from determination of the best moment for planting (Salinas et al., 2022), to fruit thinning (Salinas et al., 2017) to active control of the environmental conditions in the structures (Salinas et al., 2021). The same research group also defined thermal requirements for growth of the papaya fruit in subtropical conditions of south-eastern Spain (Salinas et al., 2019). In the same sense (Ruas et al., 2020), observed how an active climate control system can improve leaf gas exchange emphasizing the importance of mild environmental conditions for high yields and fruit quality in papaya. An interesting line of research seems to be the application of Effective Microorganisms (EM), with or without the pairing with mineral fertilizers, gave promising results in a trial in Egypt (Desoky et al., 1999).

Greenhouse cultivation of papaya offers several agronomic advantages, yet it can make such plants vulnerable to many pests and diseases. In Spain, the primary pests affecting greenhouse-grown papaya are the banana moth (*Opogona sacchari* Bojer) and the red spider mite (*Tetranychus cinnabarinus*). Among fungal pathogens, *Oidium caricae* F. Noack is the most prevalent, while *Botrytis cinerea* Pers. appears occasionally under favourable environmental conditions (Galán Saúco and Pastor, 2007). Similar pests and disease have been reported in Israel. However, a distinct condition known as Nivun Haamir Dieback (NHDB), which severely affects papaya plants, has so far been reported only in Israel (Gera et al., 2005), according to available knowledge.

Despite limited publications concerning its medicinal uses, papaya leaves, seeds, roots, and fruits are known to be widely used in African folk medicine, However, it appears relatively less commonly used across the Mediterranean region. Adel et al. (2022) reported promising antiviral activity of *Carica papaya* against SARS-CoV-2, warranting further investigation.

Recent studies on papaya have explored both postharvest strategies and cultivar-specific characteristics to enhance fruit quality, nutritional value, and shelf life, particularly under Mediterranean and greenhouse conditions. Regarding postharvest fruit quality, cold storage combined with semi-permeable packaging has proven effective in maintaining the nutritional and enzymatic integrity of fresh cut ‘Formosa’ papaya grown in Sicily (Adiletta et al., 2020). Parallel studies evaluating the performance of papaya cultivars under Mediterranean greenhouse conditions identified clear genotype-dependent differences. For instance (Farina et al., 2020b), reported that ‘Cartagena’ and ‘Maradol’ exhibited the highest levels of vitamins, polyphenols, and antioxidant activity, along with favourable sensory profiles. Similarly (Kafkas et al., 2012), highlighted cultivar variability in Turkey, with ‘Sunrise Solo’ emerging as the sweetest and richest in total soluble solids (TSS), supported by high levels of polyunsaturated fatty acids.

In comparative analyses of Turkish-grown varieties, Sel-42 displayed significantly higher phenolic content and antioxidant capacity than ‘Tainung’, suggesting its superiority for commercial exploitation (Kelebek et al., 2015). Meanwhile, Pinillos et al. (2017) investigated the optimal harvest stage for three cultivars in south-eastern Spain, concluding that harvesting at intermediate maturity ensured better TSS levels, firmness, and consumer appeal, especially for the ‘Siluet’ cultivar.

Papaya is also commercially known for its juice. Özkan et al. (2011) demonstrated that ‘Sunrise Solo’ juice had the highest antioxidant capacity among ‘Red Lady’ in respect to ‘Tainung’, with a strong correlation between total phenolic content and antioxidant activity, reinforcing the role of phenolics in juice bioactivity. Together, these findings emphasize the potential of papaya as a high-value tropical crop adaptable to Mediterranean climates. They highlight the importance of cultivar selection, harvest timing, and postharvest handling in maximizing both fresh and processed product quality. Despite its promising agronomic performance under Mediterranean conditions, papaya remains relatively underrepresented in the literature. This may be partly explained by the fact that its cultivation is largely confined to greenhouse systems, which limit production scalability, increase production costs, and may reduce its market presence and consumer familiarity compared to other tropical fruits. Consequently, these constraints may contribute to the lower research attention and slower adoption of this crop in the region.

### 4.4 Guava

Guava is largely used in traditional medicine in tropical and subtropical areas. Guava is largely used in traditional medicine in tropical and subtropical areas as well as in the Mediterranean basin. In fact, guava leaves appear to be commonly used in ethnomedicine across several Arabic Mediterranean countries. For instance, in Palestine boiled leaves are boiled and leaves are used as decoction/antimicrobial and antidiarrheal activities (Jaradat et al., 2016), in Algeria leaves are used as traditional medicine for treating diarrhoea, gastrointestinal and respiratory disturbances, hypertension, diabetes mellitus and hepatitis (Saber et al., 2018), but also for symptomatic treatment of Alzheimer disease (Bouchoukh et al., 2019).

Recent studies across the Mediterranean region have increasingly focused on the complex pest and pathogen pressures limiting guava production, with particular emphasis on biological control strategies. In Egypt, *Aspergillus versicolor* and *Bacillus subtilis* have demonstrated notable antifungal activity against anthracnose, highlighting their potential as biocontrol agents (Abdul Wahid, 1999). Similarly, natural enemies such as *Gyranusoidea indica* and *Scymnus syriacus* have proven effective in managing mealybug populations (Adly et al., 2016). Guava fruits are also heavily infested by *B. zonata* and *C. capitata*. In Syria, *Aganaspis daci* (Weld) (Hymenoptera: Figitidae) has been identified as a highly promising parasitoid for the control of *C. capitata* (Ali et al., 2015, 2016).

Interestingly, this larval-pupal parasitoid was unexpectedly recovered in Southern Italy, where it had not been intentionally released (Bernardo et al., 2023). In this context, IPM approaches in Egypt advocate the combination of biological control with complementary techniques such as “attract and kill” supported by meteorological data and degree-day models to anticipate pest emergence and enhance the precision of control measures (Bayoumy et al., 2021).

Being climacteric, guava fruits are prone to several post-harvest issues. To enhance fruit preservation and shelf life (Lo’ay and Taher, 2018), showed that edible coatings of chitosan/PVP and M salicylic acid effectively reduced browning and preserved quality by inhibiting degradative enzymes. Complementarily (Porat et al., 2009), reported that reported that 1-MCP treatment extended the shelf life of guava, particularly in newly developed cultivars such as ‘King’ and ‘Omri’, which exhibited non-climacteric ripening behaviour compared to the traditional climacteric cultivar ‘Ben Dov.’ In fact, the latter emits stronger tropical fruit odour than newly introduced varieties, which was attributed to higher levels of ester compounds (Porat et al., 2011). It’s believed volatile profiles of guavas differ by origin and pulp colour, with sesquiterpenes and sulphur compounds more abundant in Indian guava (Kamal et al., 2023). Guava waste also offers some valuable opportunities, such as the use of activated guava wood charcoal (Elsawy et al., 2023), essential oils extracted from guava leaves and stems which is rich in bioactive compounds, including Veridiflorol, β-caryophyllene, and D-limonene (Khadhri et al., 2014; Hassan et al., 2021). Despite its high nutritional and medicinal value, guava remains underrepresented in agronomic research within the Mediterranean. Although initial studies have highlighted the potential of by-products such as leaves and processing residues, this research area remains underdeveloped. Expanding investigations on the valorisation of guava by-products represents a key opportunity to enhance the economic value and sustainability of this crop in the region. Considering the high nutritional and value and medicinal properties of Guava, it would be worth it to invest more research effort on the aspects of its cultivation and environmental adaptation in the Mediterranean area.

### 4.5 Litchi

As for guava also litchi presented medicinal properties with a significant number of bioactive polyphenols, recent findings report for the first time the effects of Sicilian litchi fruit extracts on colon cancer cells, as litchi exocarp, mesocarp and endocarp showed a capability in reducing the viability and growth of HT29 cell (Emanuele et al., 2018). Such findings are not far from other research that shows that litchi extracts display anti-tumour and pro-apoptotic effects *in vitro*.

On the agronomic level, pollination is considered a major constraint in litchi cultivation across the Mediterranean as reported by several studies conducted in Israel (Stern and Gazit, 1996). Strategies to improve pollination include either by the inclusion of more cross-pollination cultivars, especially Hong Long (Raz et al., 2022). To overcome low pollination (Stern and Gazit, 1998), suggested the prioritizing of M2 pollen in future breeding due to superior viability and fruit less sensitivity to high temperature compared to M1 pollen. Recent findings also found that adding bumblebees hives in addition to honeybees can highly improve fruit set and yield (Germain et al., 2007; Sapir et al., 2019). From a pest management perspective, a first report of *Aceria litchi* was recently reported for the first time in Sicily and in Europe (Giordano et al., 2024).

Recent research on litchi fruit quality and post-harvest handling has explored innovative approaches, offering viable alternatives to traditional chemical treatments (Farina et al., 2017, 2018). One promising technique is hot water brushing (HWB), which replaces sulphur dioxide (SO_2_) fumigation, commonly used to retain the red colour of litchi but associated with health risks (Lichter et al., 2000). In addition to physical treatments, hormonal regulation through synthetic cytokinins such as CPPU has shown notable effects on litchi fruit development and storability. CPPU delayed fruit maturity by 2–3 weeks, resulting in fruits that were 20–25% larger than untreated ones and richer in soluble solids. These treated fruits also exhibited superior postharvest performance, maintaining good flavour, reduced browning, and lower decay rates during cold storage (1 °C) for up to six weeks (Stern et al., 2006). Future studies on litchi should focus on assessing its physiological behaviour and adaptation under the Mediterranean climate conditions. The limited number of studies reflects their recent introduction and restricted cultivation area in the Mediterranean. Current research is fragmented and often preliminary, highlighting a clear need for more systematic investigations into their agronomic performance, adaptability, and economic potential. Future studies on litchi should focus on assessing its physiological behaviour and adaptation under the Mediterranean climate conditions.

### 4.6 Dragon fruit

Dragon fruit, also commonly referred to as Pitaya, has recently gained popularity among consumers and small farmers, occupying a growing niche in Europe’s exotic fruit market. Its ornamental value, low water requirements, and adaptability to climate change make it a promising crop for the Mediterranean area. However, key challenges remain, particularly in its propagation, cultivation and its physiological requirements (Trivellini et al., 2020). As plant propagation is expanding, careful virus screening should be prioritized, especially for recently reported pathogens such as *Virus X* in Spain (Janssen et al., 2022). Preliminary studies in Israel show that climbing cacti are susceptible to high solar irradiation and become bleached when grown open air; instead, plants develop well and flower when they are shaded (Raveh et al., 1998). Most species needed cross-pollination for good fruit set (except self-fruitful *H. undatus*), while *S. megalanthus* was self-fruitful and could set fruit without pollinators. Cross-pollination increased fruit size, and open pollination by honeybees was less effective than hand pollination in Hylocereus but effective in *S. megalanthus* (Weiss et al., 1994). In Israel, pitaya fruits were found to reach their physiological maturity approximately 28 days after flowering growing under greenhouse in Negev desert (Nerd et al., 1999). However, aside from Israel, the distribution of pitaya cultivation in Europe is still limited, but it is gradually expanding in Spain, Portugal, Italy and Greece. For instance, pitaya is grown on the mainland (near Athens) and on the island of Rhodes, mainly by small producers. Spain is the largest pitaya producer in Europe with production concentrated in the southern region, in the Community of Andalusia (Trindade et al., 2023).

### 4.7 Passionfruit

A similar trend and pattern were observed in *Passiflora edulis* (Sims.), which has gained significant attention both for its edible fruit and as an ornamental evergreen climber. In addition, its chemical composition is used in the pharmaceutical industry, particularly due to the glycoside passiflorine as sedative or tranquilizing properties. Beyond that, passionfruit has gained popularity in the Mediterranean region of Turkey. A recent study by (Ada et al., 2024), showed that both *P. edulis* and *P. caerulea* exhibited high antioxidant activity.

In recent years, there have been several reports of viruses on Passionfruit. This can be due to the plant being propagated mainly by vegetative cutting, which facilitates the spread of viral infections. Throughout nucleotide sequencing two recently discovered viruses *Passiflora edulis* symptomless virus (PeSV) and *Passiflora chlorosis* virus (PaCV) in passion fruit plants cultivated in Israel (Jover-Gil et al., 2018; Fresnillo et al., 2022). Earlier in 2008 *Cucumber mosaic virus* was isolated from a passion fruit plant in southern of Italy (Parrella and Sorrentino, 2009). Few years later, PeSV has also been detected in pomegranate leaves in Spain, suggesting a broader host range or potential cross-species transmission. Only one study conducted in Sicily, Italy, covers the incidence of *Fusarium nirenbergiae* (from the *Fusarium oxysporum* species complex) (Aiello et al., 2021) which causes wilting of the passionfruit plant. Further studies are needed for both species to better understand their adaptability, geographical range, economic potential, nutritional importance, and susceptibility to pests and diseases.

### 4.8 Cherimoya

Cherimoya can still be considered a minor fruit due to the limited area harvested. However, Spain hosts the largest germplasm bank collection worldwide of Annona specimens with over 350 accessions. Such collection represents a valuable resource and plays a key role in preserving genetic diversity, future conservation strategies, and materials for new varietal selections.

Several genetic studies were performed on this collection using different approaches and genetic markers including; isoenzymatic markers (Perfectti and Pascual, 1998), SSR (Escribano et al., 2007) and DNA barcoding (Larranaga et al., 2022). These studies highlight the presence of different genetic similarity cases: Fino de Jete and Hill, Campas and Campas mejorada, and Concha, Concha lisa and Azapa-II. Also, the presence of two distinct haplotypes with geographic associations was detected. Haplotype 1 is present all over the cherimoya distribution area, while haplotype 2 is characteristic of Central America, mainly Honduras (Larranaga et al., 2022). These results confirm the efficiency of genetic tools for identifying intraspecific diversity and guiding *ex situ* conservation, thus allowing them to detect desirable traits for cherimoya cultivation in the Mediterranean climate.

A Spanish research team investigated the reproductive biology of the main cultivated variety of cherimoya in Spain by exploring pre- and post- pollination reproductive barriers. Wind does not play any role in cherimoya pollination and pollinizer insects were found to be inefficient in transferring pollen grains. Cherimoya flowers do not reject self-pollen to achieve fertilization. *A. cherimola* shows preferential allogamy based on efficient dichogamy reinforced by elevated synchrony among flowers in their sexual phases. Herkogamy, instead, hampers autogamy, although pollen deposition by gravity in cherimoya pendulous flowers explains the reduced reproductive success observed in isolated flowers (González and Cuevas, 2011). Another study (González and Cuevas, 2008) investigated the frequency, fertility and flowering dates and patterns of different kind of shoots (high, medium and low vigour) in trees of the cv (González and Cuevas, 2011). Another study (González and Cuevas, 2013) investigated the frequency, fertility and flowering dates and patterns of different kind of shoots (high, medium and low vigour) in trees of the cv. Fino de Jete: the results shows that low shoot vigour induces lower flower production.

Studies that fell into the “Crop management” topic were found to investigate cultural techniques applied in small scale trials. In particular, researchers found that cherimoya trees in a Mediterranean environment benefit from mulching (García-Carmona et al., 2020), foliar application of micro-elements (González et al., 2008; Hammoud et al., 2022) and manual pollination (Caleca, 1996; Farré et al., 1999; Caleca et al., 2002) to make up for the lack of effective natural pollinizers in the area (González and Cuevas, 2011).

Irrigation management of cherimoya was studied in several countries. In Lebanon (El-Beltagi et al., 2019), compared drip and mini-sprinkler irrigation on *Annona squamosa* L. plants, showing that drip irrigation significantly improved vegetative growth, root development, and fruit yield compared to mini-sprinklers. In southeastern Spain, studies on cherimoya cv. Fino de Jete reported crop coefficient (Kc) values of 0.63 (flowering), 0.68 (fruit set), and 0.55 (fruit growth), with high yields in both conventional and organic systems (44–47 kg tree^-^¹) and slightly higher water-use efficiency under conventional management (Durán Zuazo et al., 2019). Soil studies revealed that cherimoya residues decomposed more rapidly than other subtropical crops, releasing over 70% of their initial nitrogen during the study period, thus contributing efficiently to short-term nitrogen cycling (Rodríguez Pleguezuelo et al., 2009). However the same researchers point out nutrient leaching risks in cherimoya orchards especially after heavy rainfall, with concentrations in drainage water occasionally exceeding safe thresholds (Rodríguez Pleguezuelo et al., 2011). Overall, cherimoya proves highly productive and contributes to short-term soil fertility, but efficient irrigation and fertilization management are essential to reduce nutrient losses and ensure long-term sustainability in Mediterranean systems.

Cherimoya trees are susceptible to several serious diseases that negatively affect yield and fruit quality. However, only a single study was found addressing pathogens on cherimoya fruits in the Mediterranean. In fact (Haggag and Nofal, 2006), reported *Botryodiplodia theobromae* as a serious pathogen affecting cherimoya trees in Egypt, causing dry rot and the dieback of flowers, fruits, and branches. Their study showed that applying multiple control bioagents, including *Trichoderma koningii* and *T. hamatum*, significantly reduced disease incidence and improved fruit yield. Importantly, the combined bioagents were more effective than any single or dual treatment.

As for the post-harvest life of the fruit, cherimoya responded well to a trial of active Modified Atmosphere Packaging (MAP) which allowed it to delay the reaching of optimal consumption point compared to untreated fruits (Tinebra et al., 2022).

Researchers in few countries have obtained interesting and promising results on cherimoya, which would justify the diffusion of the crop in more areas than those where it is cultivated now. Overall, although cherimoya demonstrates high agronomic potential and contributes positively to soil fertility and productivity under Mediterranean conditions, the available research remains relatively limited and fragmented compared to other tropical crops. Despite the existence of extensive germplasm resources, their effective integration into breeding programs and cultivation strategies remains insufficiently explored. This highlights the need to better exploit the available germplasm resources, using these genetic materials more effectively in breeding programs and varietal selection to support the long-term development of cherimoya.

## 5 Future perspectives

This review serves as both a compendium and a baseline for future research on tropical fruit cultivation in the Mediterranean. To improve the future status of this sector, several recommendations are provided addressing current knowledge gaps, sustainable practices, and collaborative opportunities. Further studies are recommended to fill the knowledge gaps identified for each of the studied fruits, particularly those that are least studied. Moreover, it is strongly suggested to focus on the adaptation of these fruits across understudied areas, including the development of concrete strategies to mitigate environmental factors.

Future research should focus more on plant adaptation through advanced water management studies, especially by combining it with the physiological responses of plants under water stress conditions. In addition, emphasis should be placed on developing more sustainable agricultural practices, which could support non-EU Mediterranean countries in meeting EU standards. This would enable them to export their tropical fruits to the European market, thereby increasing economic value and strengthening self-sufficiency across the region. While pest and pathology studies are currently the most common, more in-depth research on practical treatments is advocated.

Furthermore, the use of available databases should be expanded to include predictive studies, including the modelling of potential distribution and suitability of economically important pests, as well as the early identification of species that may pose future risks. The presence of several local varieties represents a valuable resource for future *in situ* and *ex situ* conservation, as well as for the development of breeding programs. Expanding the use of these local varieties is particularly relevant, as they often demonstrate better suitability to local biotic and abiotic factors. The expansion into international markets requires greater research attention on post-harvest practices, particularly regarding storage, transportation, and shelf-life extension of the fruits. Optimizing these practices can reduce losses, maintain quality, and enhance the competitiveness of Mediterranean fruits in both local and global markets. To meet all this perspective and goals we are delighted to introduce TropMed, an initiative establishing a Mediterranean-based platform to strengthen collaboration among researchers working on tropical and subtropical fruits across the Mediterranean basin. This network is designed to connect researchers, institutions, and disciplines. With this platform, we aim to enhance future collaborative work, stimulate connections and set future policies between researchers involved in the study of this fast-growing sector. We invite colleagues to contribute by submitting their data through the following online platform: [https://docs.google.com/spreadsheets/d/1oa86l7JXGexYDrSFnD197np-befjmZVGVQxHZBdc8Aw/edit?gid=0#gid=0]. This resource is intended as a dynamic and continuously updated database within the framework of the TropMed initiative.

## Data Availability

The original contributions presented in the study are included in the article/**Supplementary Material**. Further inquiries can be directed to the corresponding authors.
