# Supplementary material for: Tropical fruits in the Mediterranean Basin: current research status, priorities, and knowledge gaps. A systematic review
Source: Front Plant Sci. 2026 Jun 5;17:1817537. doi: 10.3389/fpls.2026.1817537 (PMC13278884; doi:10.3389/fpls.2026.1817537)
Supplement: Supplementary file 1 [file DataSheet1.pdf]

Title	Species	Var1	Var2	Var3	Var4	Var5	Var6	Var7	Var8	Var9	Var10	Var11	Var12	Var13	Var14	Var15	Var16	Var17	Var18	Var19	Var20	Var21	Var22	Var23	Var24	Var25	Var26	Var27	Var28	Var29	Var30	Var31	Var32
Air Temperature as a Key Indicator of Avocado (Cvs. Fuerte, Zutano, Hass) Maturation Time in Mediterranean Climate Areas: The Case of Western Crete in Greece	Avocado	Hass	Fuerte	Zutano																													
Chemical characterization of oil from four Avocado varieties cultivated in Morocco	Avocado	Ettinger	Fuerte	Hass	Reed																												
Chemical composition, bioactive compounds and antioxidant activity of six avocado cultivars Persea americana Mill. (Lauraceae) grown in Egypt	Avocado	Hass	Ettinger	Pinkerton																													
Comparing 'Iriet' and 'Ettinger' avocado cultivars as pollinators of 'Hass' using SNPs for paternal identification	Avocado	Hass	Iriet	Ettinger																													
Correlation between genetic variability, chemical composition and antimicrobial activity of essential oils isolated from avocado cultivars grown in Egypt	Avocado	Bacon	Duke	Ettinger	Pinkerton																												
Outcrossing rate, yield, and selective fruit abscission in 'Ettinger' and 'Ardith' avocado plots	Avocado	Ettinger	Ardith																														
Evaluation of Postharvest Maturity Indices of Commercial Avocado Varieties Grown at Various Elevations Along Lebanon's Coast	Avocado	Hass	Lamb Hass	Ettinger	Fuerte	Pinkerton	Reed	Horshim																									
First record of the Persea Mite Oligonychus perseae (Acari: Tetranychidae) in Italy with a review of the literature	Avocado	Bacon	Hass																														
Preliminary assessment of maturity and picking dates of avocado under Lebanese growing conditions	Avocado	Hass	Lamb Hass	Pinkerton	Reed																												
Postharvest use of organic coating for maintainings bio-organic avocado and mango quality	Avocado	Ettinger																															
Postharvest fungicide for avocado fruits: Antifungal efficacy and peel to pulp distribution kinetics	Avocado	Pinkerton	Ettinger	Reed																													
Quantitative evaluation of the phenolic profile in fruits of six avocado (Persea americana) cultivars by ultra-high-performance liquid chromatography-heated electrospray-mass spectrometry	Avocado	Hass	Bacon	Fuerte	Pinkerton	Rincon	Orotawa																										
DETECTION OF AVOCADO SUNBLOTCH VIROID IN SPAIN BY DOUBLE POLYACRYLAMIDE-GEL ELECTROPHORESIS	Avocado	Hass	Fuerte																														
Effect of Organic Fertilizers on Avocado Trees (Cvs. Fuerte, Hass, Lamb Hass) in Western Crete, a Cool Subtropical Region	Avocado	Fuerte	Hass	Lamb Hass																													
Effect of soil solarization on the control of Phytophthora a root rot in avocado	Avocado	Fuerte	Pinkerton																														
Determination of the irrigation regimen for an avocado plantation in spring and autumn	Avocado	Hass	Ettinger	Fuerte																													
Effect of pre-incubation humidity and temperature treatment on the in vitro germination of avocado pollen grains	Avocado	Fuerte	Nabal	Ettinger	Bacon	Zutano																											
Effect of soil solarization on the control of Phytophthora root rot in avocado	Avocado	Fuerte	Pinkerton																														
The performance of some avocado cultivars under Mediterranean coastal conditions in Turkey	Avocado	Bacon	Clifton	Corano	Edranol	Ettinger	Fuerte	Hass	Nowels	Pinkerton	Reed	Regina	Rincon																				
The time of flowering of avocado and the female and male opening of flowers in Crete	Avocado	Topa-Topa	Mexicola	Yama	Duke	Condor	Fuerte	Ettinger	Mexico	Rincon	Bacon	Zutano	Benik																				
Tree Growth and Fruit Quality of Twenty Avocado Cultivars in Cyprus	Avocado	Pinkerton	Fuerte	Ettinger	Hass	Arturo	Tova	Horshim	Fucks	Maoz	Nachlat	Nabal	Reed																				
Variation in the composition of Cretan avocado cultivars during ripening	Avocado	Ettinger	Fuerte	Hass																													
The role of Euwallacea nr. fornicatus (Coleoptera: Scolytinae) in the wilt syndrome of avocado trees in Israel	Avocado	Hass	Ettinger	Ardit	Fuerte	Galil	Nabal	Fino	Pinkerton	Reed	Horshim																						
Water requirements of avocado in Israel. I. Tree and soil parameters†	Avocado	Hass	Fuerte	Ettinger																													
Water requirements of avocado in Israel. II.* Influence on yield, fruit growth and oil content†	Avocado	Hass	Fuerte	Ettinger																													
Within orchard spatial distribution of mature avocado trees mortality	Avocado	Hass	Reed	Ettinger																													
Low attractiveness of avocado (Persea americana Mill.) flowers to honeybees (Apis mellifera L.) limits fruit set in Israel	Avocado	Hass	Ettinger	Reed	Nabal																												
Influence of physical distance between cultivars on yield, outcrossing rate and selective fruit drop in avocado (Persea americana, Lauraceae)	Avocado	Hass	Fuerte																														
Selection of potential pollinizers for 'Hass' avocado based on flowering time and male-female overlapping	Avocado	Hass	Fuerte																														
Seasonal changes of lipids and fatty acids in two varieties of Avocado pear fruits	Avocado	Fuerte	Deuke																														
Selection and breeding of honey bees for higher or lower collection of avocado nectar	Avocado	Hass	Ettinger	Pinkerton	Fuerte																												
Seven Persea americana varieties essential oils comparison: Chemical composition, toxicity, antibacterial, and antioxidant activities	Avocado	Ettinger	Fuerte	Hass	Reed	Zutano	Bacon	Maluma Hass																									
The effect of storage temperature on the quality of avocado fruits from different climatic zones	Avocado	Hass	Fuerte																														
Flowering behaviour of 19 avocado cultivars in Crete	Avocado	Fuerte	Bacon	Nabal	Hass	Rincon	Anaheim	Reed																									
Flower development and timing of avocado (Persea americana) growing under Tunisian conditions	Avocado	Hass	Fuerte	Bacon																													
Fruit development effect on fatty acid composition of persea americana fruit mesocarp	Avocado	Lula	Bacon	Fuerte	Zutano																												
A Study of the Effect of Replacing Drip Irrigation System by Micro-Sprinklers in Avocado (Persea americana Mill) in Morocco	Avocado	Hass	Fuerte	Zutano	Bacon																												
Adaptation and performance of 15 avocado cultivars grown in Antalya Province in southern Turkey	Avocado	Fuerte	Hass	Bacon	Zutano	Wurtz	Reed	Ryan	Santana	Ettinger	Pinkerton	Corona	Rincon																				
WATER-USE, WETTED SOIL VOLUME, ROOT DISTRIBUTION AND YIELD OF AVOCADO UNDER DRIP IRRIGATION	Avocado	Fuerte																															
Comparative study of some fruit quality characteristics of two of Annona cherimola Mill. grown in southern Italy	Cherimoya	Fino de Jete	Campas																														
Monitoring the pollution risk and water use in orchard terraces with mango and cherimoya trees by drainage lysimeters	Cherimoya	Fino de Jete																															
Development of an Effective Sonotrode Based Extraction Technique for the Recovery of Phenolic Compounds with Antioxidant Activities in Cherimoya Leaves	Cherimoya	Fino de Jete	Negrio joven	Campas-1	Campas-2																												
Yield, pomological characteristics, bioactive compounds and antioxidant activity of Annona cherimola Mill. grown in mediterranean climate	Cherimoya	Fino de Jete	d Torre 1	Totapuri	Baladi Dabsha																												
Improving the biological control of Botryodiplodia disease on some Annona cultivars using single or multi-bioagents in Egypt	Cherimoya	Balady	Abd-El-Razik	Hindy																													

Title	Species	Var1	Var2	Var3	Var4	Var5	Var6	Var7	Var8	Var9	Var10	Var11	Var12	Var13	Var14	Var15	Var16	Var17	Var18	Var19	Var20	Var21	Var22	Var23	Var24	Var25	Var26	Var27	Var28	Var29	Var30	Var31	Var32
RESEARCH ON THE SUBTROPICAL FRUITS PRODUCED IN CALABRIA - THE CHERIMOYA (ANNONA-CHERIMOLA, MILL)	Cherimoya	Claudia	Anna	Bettina	Daniela	Elena																											
Identification and quantification of phenolic and other polar compounds in the edible part of Annona cherimola and its by-products by HPLC-DAD-ESI-QTOF-MS	Cherimoya	Campa	Fino de Jete																														
Aroma volatile compositions of high- and low-aromatic guava varieties	Guava	King	Lior	Ben Dov	z	Roni																											
Comparative Chemical Profiles of the Essential Oils from Different Varieties of Psidium guajava L.	Guava	Red Malaysian	El-Qanater	White Indian	Early	El-Sabahya El-Gedida	Red Indian																										
Postharvest Longevity and Responsiveness of Guava Varieties with Distinctive Climacteric Behaviors to 1-Methylcyclopropene	Guava	Ben Dov	King	Omri																													
Aroma-based discrimination of Egyptian versus Indian guava fruits and in response to probiotics as analyzed via SPME/GC-MS and chemometric tools	Guava	Pomifera	Pyrifera	Bullock's Heart	Company	Dabsha	Green Succari	Hindi Mouloky	Hindi Sennara	Nabeel	Succari	Zebda																					
Pollen viability in lychee	Litchi	Mauritius	No Mai Chee	Wai Chee	Early Large Red	Floridian																											
Woody Canker and Shoot Blight Caused by Botryosphaeriaceae and Diaporthaceae on Mango and Litchi in Italy	Litchi	Way Chee	Kwai Mai Pink																														
THE EFFECT OF 3-5-6 TPA ON FRUIT DROP AND FRUIT SIZE IN THE LYCHEE (LITCHI CHINENSIS) CULTIVARS 'FAY ZEE SIU' ('FEIZIXIAO'), 'KAIMANA', 'KWAI MAI PINK', 'SOUEY TUNG' AND 'TAI SO' ('MAURITUS')	Litchi	Fay Zee Siu	Kaimana	Kwai Mai Pink	Souey Tung	Tai So																											
Fruit production and quality evaluation of four litchi cultivars (Litchi chinensis Sonn.) grown in Mediterranean climate	Litchi	Tai So	Wai Chee	Brewster	Kwai Mai																												
Hong Long Lychee (Litchi chinensis Sonn.) Is the Optimal Pollinizer for the Main Lychee Cultivars in Israel	Litchi	Mauritius	Fei Zi Xiao	Tamuz	Hong Long																												
Hong Long Lychee (Litchi chinensis Sonn.) Is the Optimal Pollinizer for the Main Lychee Cultivars in Israel	Litchi	Hong Long	Nuomici	Fay Zee Siu	Mauritius	Tamuz																											
Supplementing bumblebees to 'Mauritius' lychee improves yield	Litchi	Mauritius																															
Different factors involved in the low fruit set of mango (Mangifera indica)	Mango	Kent	Keitt	Kensington Pride	Osteen																												
Reproductive biology of mango (Mangifera indica) in a Mediterranean climate	Mango	Kent	Keitt	Kensington Pride	Osteen	Stewart	Wurtz	Zutano	Benedict	Gottfried	Jalna	Jerma	Jim	Lula	Puebla	Ryan	Santana	Sharwil	Shawn T.6	Simpson	SRA-2	Susan											
Return Analysis of 'Osteen' and 'Kent' Mango Orchards in South-Eastern Mainland Spain	Mango	Osteen	Kent	Mac Arthur	Hass	Dickinson	Booth	Anaheim	Reed	Nabal																							
Biodiversity of mites in mango orchards (Mangifera indica L.) and evaluation of some mineral and essential oils against Cisaberoptus kenyae Keifer (Acari: Eriophyidae) management	Mango	Hindi	Zebda	Alphonso	Evans	Sedika																											
Carotenoid content and pulp colour non-destructively measured by time-resolved reflectance spectroscopy in different cultivars of Brazilian mangoes	Mango	Haden	Palmer	Shomrat	Whitsel	Benik	No. 2631	Netain	Topa-Topa	Bacon	Lula																						
Characterization of Fusarium mangiferae isolates from mango malformation disease in Southern Spain	Mango	Osteen	Tommy Atkins	Keitt	Dusheri	Otts	Kent	Haden																									
Characterization of Pseudomonas syringae pv. syringae isolated from mango in Sicily and occurrence of copper-resistant strains	Mango	Kensington Pride	Osteen	Tommy Atkins																													
COMPARATIVE STUDY ON FRUIT YIELD AND QUALITY TRAITS OF THE NEW MANGO CULTIVARS GROWN UNDER EGYPT CONDITIONS	Mango	Kent	Palmer	Yasmina Rose	Shelly	Nam Dok Mai	Osteen	Glenn	Sensation	Kensington Pride	Heidi	Joa																					
Comparative study on the quality characteristics of some Egyptian mango cultivars used for food processing	Mango	Alphonso	Sedeka	Awis	Sinara	Sukari	Zibdia																										
Microbiome Alterations Are Correlated with Occurrence of Postharvest Stem-End Rot in Mango Fruit	Mango	Shelly																															
Monitoring the pollution risk and water use in orchard terraces with mango and cherimoya trees by drainage lysimeter	Mango	Osteen																															
Multifaceted Health Benefits of Mangifera indica L. (Mango): The Inestimable Value of Orchards Recently Planted in Sicilian Rural Areas	Mango	Kensington Pride	Keitt	Glenn	Maya	Tommy Atkins																											
Environmentally friendly treatment alternatives to Bordeaux mixture for controlling bacterial apical necrosis (BAN) of mango	Mango	Tommy Atkins	Keitt																														
Epidemiological aspects of mango malformation disease caused by Fusarium mangiferae and source of infection in seedlings cultivated in orchards in Egypt	Mango	Keitt	Sucharri																														
Evaluation of quality attributes and consumer preference of fresh or imported mangoes in Italy	Mango	Keitt	Kent	Osteen																													
First record of Icerya seychellarum and confirmed occurrence of Aulacaspis tubercularis (Hemiptera: Coccomorpha) in Italy	Mango	Glenn	Keitt	Valencia Pride	Osteen	Tommy Atkins	Irwin																										
First Report of Mango Malformation Disease Caused by Fusarium mangiferae in Spain	Mango	Keitt	Kent	Osteen	Tommy Atkins																												
Preliminary investigation of Verticillium wilt on mango trees (Mangifera indical.) in Egypt	Mango	Keitt	Zebda																														
Postharvest use of organic coating for maintainings bio-organic avocado and mango quality	Mango	Tommy Atkins																															
Phenology and Fruit Growth Dynamics of Mango (Mangifera indica L.) in Greenhouse and Open Air in Mediterranean Climate	Mango	Tommy Atkins	Osteen	Keitt																													
Phenological cycle of three mango cultivars in the Mediterranean climate	Mango	Keitt	Osteen	Tommy Atkins																													
Phenology and management of the white mango scale, Aulacaspis tubercularis Newstead (Hemiptera: Diaspididae), in Southern Spain	Mango	Osteen	Keitt																														
Physico-chemical Quality Parameters of Mango (Mangifera indica L.) Fruits Grown in a Mediterranean Subtropical Climate (SE Spain)	Mango	Tommy Atkins	Lippens	Sensation	Osteen	Irwin	Gleen	Kensington Pride	Valencia Pride	Palmer																							
Phytochemical and antimicrobial investigation of the leaves of five Egyptian mango cultivars and evaluation	Mango	Alphonso	Sidik	Ewase	Zebda	Fagri-kalan																											
Physiological Behaviour of Mangos with Different Ploidy Levels	Mango	Blanca	Fina	Turpentine																													
Phytochemical Differentiation among Fourteen Cultivars of Mango (Mangifera indica L.) Leaves from Al-Sharqia, Egypt	Mango	Keitt	Ewais	White Succari	Tommy Atkins	Fajri Kalan	Zebda	Alphonso	Sedeek	Naomi	Mesk	Baladi Arnaba	Cobania																				
Population fluctuation of some economically important mites on two mango cultivars in Qalyubia governorate, Egypt	Mango	Heidi	Naomi																														
Current status and impact of mango malformation in Egypt	Mango	Company	Taimour	Pairi	Mabrouka	Alphonse	Fisher	Awais	White succari	Baladi	Lux	Mesk	Hindi baracot																				
Drying kinetics and physico-chemical quality of mango slices	Mango	Keitt	Osteen																														
Tracing the geographical origin of Spanish mango (Mangifera indica L.) using stable isotopes ratios and multi-element profiles	Mango	Osteen	Kent	Palmer	Keitt	Irving																											
Tree-Ripe mango fruit: Physicochemical characterization, antioxidant properties and sensory profile of six mediterranean grown cultivars	Mango	Keitt	Glenn	Osteen	Maya	Kensington Pride	Tommy Atkins																										

Title	Species	Var1	Var2	Var3	Var4	Var5	Var6	Var7	Var8	Var9	Var10	Var11	Var12	Var13	Var14	Var15	Var16	Var17	Var18	Var19	Var20	Var21	Var22	Var23	Var24	Var25	Var26	Var27	Var28	Var29	Var30	Var31	Var32
Woody Canker and Shoot Blight Caused by Botryosphaeriaceae and Diaporthaceae on Mango and Litchi in Italy	Mango	Kent	Keitt	Sensation	Osteen	Kensington Pride																										
Impact of treated wastewater and salicylic acid on physiological performance, malformation and yield of two mango cultivars	Mango	Keitt	Ewais																													
In vitro production of somatic embryos from nucellus of mango (Mangifera indica L.)	Mango	Zebda	Sedeek	Hind																												
Management of mango malformation disease based on a novel strategy of timing of fungicide applications combined with sanitation	Mango	Maya	Keitt	Tommy Atkins																												
Integrated management of powdery mildew of mango in Egypt	Mango	Alphonso	Seddek	Zebda	Hendi Besenara	Ewise																										
Influence of Pre-Harvest Bagging on the Incidence of Aulacaspis tubercularis Newstead (Hemiptera: Diaspididae) and Fruit Quality in Mango	Mango	Osteen	Sensation																													
Mango (Mangifera indica L.) germplasm diversity based on single nucleotide polymorphisms derived from the transcriptome	Mango	Keitt	Tommy Atkins																													
Reproductive biology of mango (Mangifera indica) in a Mediterranean climate	Mango	Kent	Keitt	Kensington Pride	Osteen																											
Return Analysis of 'Osteen' and 'Kent' Mango Orchards in South-Eastern Mainland Spain	Mango	Osteen	Kent																													
Screening of phenolic compounds in by-product extracts from mangoes (Mangifera indica L.) by HPLC-ESI-QTOF-MS and multivariate analysis for use as a food ingredient	Mango	Keitt	Sensation	Gomera 3																												
The acclimatisation of new tropical and subtropical fruit trees in Palestine	Mango	Avocado	Persimmon	Annona	Loquat	Papaya	Passion Fruit	Minor Fruits																								
First Report of Verticillium Wilt Caused by Verticillium dahliae on Mango Trees (Mangifera indica) in Southern Spain.	Mango	Kent	Osteen																													
Folic Acid Intake and Neural Tube Defects: Two Egyptian Centers Experience	Mango	Carrie	Keitt	Glenn	Manzanillo	Maya	Rosa	Osteen	Tommy Atkins	Kensington Pride																						
Fruit quality and photosynthetic response of three cultivars of mango (Mangifera indica L.) in a greenhouse in south of Italy	Mango	Keitt	Osteen	Tommy Atkins																												
Fruit yield, growth and leaf-nutrient status of mango trees grafted on two rootstocks in a marginal growing area (South-East Spain)	Mango	Gomera 1	Gomera 3	Osteen	Keitt																											
Genetic and Morphological Characterization of Mangifera indica L. Growing in Egypt	Mango	Yasmina	Golek	Alphonso	Piva	R2E2	Sabre	Heidi	Osteen	Langra	Benersi	Maya	Nam Dok Mai	Hindi	Mloki	Fajri	Kalan	Sidik	Joa	Sensation	Tommy Atkins	Kent	Haden	Naomi	Palmer	Shelly	Kensington Pride	Lilly	Succari	Hindi	Besennara	Zebda	Princess
Hoverfly pollination enhances yield and fruit quality in mango under protected cultivation	Mango	Osteen	Irwin	Keitt	Ataulfo	Langra																										
HPLC-DAD-Q-ToF-MS profiling of phenolic compounds from mango (Mangifera indica L.) seed kernel of different cultivars and maturation stages as a preliminary approach to determine functional and nutraceutical value	Mango	Keitt	Kent	Osteen																												
Analysis of the effect of heat stress during flowering on the yield of avocado under mediterranean climatic conditions	Mango	Hass	Nabel	Fuerte	Ettinger																											
A second Stock-Scion trial with mango in Israel	Mango	Adams	Faizanson	Gedong	Kent	Menucha	Nimrod	Pairee	Shepard	Zill	Zrifin	Alphonso	Bullock's Heart																			
Assessment of genetic diversity and relationships among Egyptian mango (Mangifera indica L.) cultivers grown in Suez canal and sinairegion using RAPD markers	Mango	Sedeek	Succary momtaz	Barbary	Alfonse	Ewais	Zebda	Succary Abiad		Sinai -1	Sinai-2	Sinai-3																				
Comparative evaluation of volatiles, phenolics, sugars, organic acids and antioxidant properties of Sel-42 and Tainung papaya varieties	Papaya	Sel-42	Tainung																													
Physicochemical, Nutraceutical and Sensory Traits of Six Papaya (Carica papaya L.) Cultivars Grown in Greenhouse Conditions in the Mediterranean Climate	Papaya	Guinea Gold	Sinta	Honeydew	Cartagena	Maradol	Solo																									
Physicochemical Characteristics and Fatty Acid Composition of Four Papaya Cultivars Grown under Plastic Greenhouse Conditions	Papaya	Red Lady	Sunrise Solo	Tainung	BH-65	Carrie	Dashehari	Edward	Gailour	Irwin	Langra	Mistikawi	Maya	Haden	Mabroka																	
Protected cultivation of 'BH-65', 'Siluer, 'Sensation', 'Intenzza' and 'Red Lady' papaya cultivars in South East Spain	Papaya	BH-65	Siluet	Sensation	Intenzza	Red Lady																										
The Production and Quality of Different Varieties of Papaya Grown under Greenhouse in Short Cycle in Continental Europe	Papaya	Intenzza	Sweet Sense	Vitale	Caballero	Alicia																										
Fruit growth model, thermal requirements and fruit size determinants in papaya cultivars grown under subtropical conditions	Papaya	BH-65	Siluet	Calimosa	Red Lady																											
Fruit quality characterization and harvest maturity index of three papaya cultivars grown in greenhouses in south-east Spain	Papaya	Intenzza	Siluet	BH-65																												
Fruit thinning in 'BH-65' and 'Intenzza' papaya grown in greenhouses	Papaya	Intenzza	BH-65																													
Growth, yield and fruit quality of three papaya cultivars grown under protected cultivation	Papaya	BH-65	SS-45	Sel-42																												
Antioxidant capacity of juice from diff erent papaya (Carica papaya L.) cultivars grown under greenhouse conditions in Turkey; [Türkiye'de serada yetiştirilen üç farklı papaya (Carica papaya L.) çeşidinden elde edilen meyve suyunun antioksidan kapasitesi]	Papaya	Sunrise Solo	Red Lady	Tainung																												
